# Mechanism-driven nano-therapeutic platform integrating plant-derived compounds and membrane-active peptides reprograms inflammatory, cell cycle arrest, cGAS–STING, and apoptotic gene expression to quench colorectal cancer

**DOI:** 10.3389/fphar.2026.1963760

**Published:** 2026-10-27

**Authors:** Doaa Ibrahim, Safaa I. Khater, Ahmed Abdelfattah-Hassan, Nouf A. Babteen, Leena S. Alqahtani, Safa H. Qahl, Mahmoud Mostafa, Noha Osama El-Shaer, Sherief M. Abdel-Raheem, Norah Alkhayyal, Randah Alghamdi, Nasiruddin Khan

**Affiliations:** 1 Department of Nutrition and Clinical Nutrition, Faculty of Veterinary Medicine, Zagazig University, Zagazig, Egypt; 2 Department of Biochemistry and Molecular Biology, Faculty of Veterinary Medicine, Zagazig University, Zagazig, Egypt; 3 Department of Veterinary Medicine, College of Applied and Health Sciences, A’Sharqiyah University, Ibra, Oman; 4 Department of Anatomy and Embryology, Faculty of Veterinary Medicine, Zagazig University, Zagazig, Egypt; 5 Department of Biological Sciences, College of Science, University of Jeddah, Jeddah, Saudi Arabia; 6 Department of Pharmaceutics, Faculty of Pharmacy, Minia University, Minia, Egypt; 7 Department of Pharmaceutics, Faculty of Pharmacy, Minia National University, Minia, Egypt; 8 Department of Physiology, Faculty of Medicine, Benha University, Banha, Egypt; 9 Department of Public Health, College of Veterinary Medicine, King Faisal University, AL‐Ahsa, Saudi Arabia; 10 Department of Health Sciences, College of Applied and Health Sciences, A’Sharqiyah University, Ibra, Oman

**Keywords:** angiogenesis, apoptosis, cell cycle, colorectal cancer, liposomal melittin peptides, niosomal chrysin, oxidative stress

## Abstract

Colorectal cancer (CRC) remains one of the leading causes of cancer-related mortality worldwide, and its first-line chemotherapeutic, 5-fluorouracil (5-FU), is constrained by poor tumor selectivity, dose-limiting systemic toxicity, and the frequent emergence of drug resistance. Nanocarrier-based co-delivery of naturally derived, multi-targeted agents offers a promising strategy to enhance efficacy while limiting off-target harm. This study evaluated the antitumor effects of nanoformulated liposomal melittin peptides (LIP-MLT) and niosomal chrysin (NsCR), administered as monotherapy or as combined therapy (LIP-MLT/NsCR), against DMH-induced CRC in a rat model. CRC was induced by subcutaneous injection of DMH (1,2-dimethylhydrazine, 40 mg/kg, twice weekly) for 8 weeks. Sprague-Dawley rats (n = 120) were randomly divided into six groups: healthy control (CON); untreated colorectal tumour (CR-TUM); and DMH-induced rats treated with 5-fluorouracil (CR-TUM + FU5), liposomal melittin (CR-TUM + LIP-MLT), niosomal chrysin (CR-TUM + NsCR), or the combination (CR-TUM + LIP-MLT/NsCR). Oxidant/antioxidant status, inflammatory cytokines, and the transcriptional profiles of inflammatory, cell-cycle-arrest, apoptotic/antiapoptotic, *cGAS*–*STING* innate-sensing, *mTOR*, and angiogenesis/metastasis genes were assessed, together with histopathological and immunohistochemical evaluation. The untreated CR-TUM group showed pronounced tumor-markers elevation, anemia, oxidative damage, and a strong pro-inflammatory, immunosuppressive shift. Relative to CR-TUM, LIP-MLT/NsCR lowered the tumor markers CEA (3.1-fold), CA19-9 (3.5-fold), and CCSA (2.1-fold), and reduced the oxidative markers ROS (2.2-fold), MDA (1.8-fold), and NO (2.2-fold) while raising the antioxidants. It also decreased the pro-inflammatory mediators NF-κB (2.3-fold), *IL*-*1*β (2.4-fold), and *TNF*-α (1.7-fold) versus CR-TUM, reversing approximately 71% of the DMH-induced inflammatory signature. At the transcriptional level, the combination produced the strongest upregulation of the pro-apoptotic genes (Caspase-3, Caspase-8, *BAX*, and *p53*) with concurrent downregulation of the antiapoptotic mediators (Bcl-2, c-*FLIP*, and Survivin), and marked proliferative and metastatic drivers suppression, including CDK4, *CDK6*, cyclin D1, β-catenin, *mTOR*, *MMP*-9, and VEGF, alongside restoration of the *CDK* inhibitors *p21*, p27, and KLF4 and normalization of cGAS, STING, and IRF3 mRNA levels. LIP-MLT/NsCR further best-preserved body weight and colon length, lowered diarrhea and rectal-bleeding scores, restored colonic histopathological architecture, and most strongly diminished the immunoexpression of *MMP*-9 and VEGF relative to untreated cancer tissues. Taken together, the combined LIP-MLT/NsCR nanoformulation attenuated DMH-induced CRC progression to a greater extent than either monotherapy or conventional 5-FU, supporting the combined melittin–chrysin nanoformulation as a promising, multi-targeted strategy against colorectal cancer.

## 1 Introduction

Despite decades of therapeutic progress, colorectal cancer (CRC) continues to claim more lives each year than almost any other malignancy, ranking among the top three causes of cancer-related death worldwide (Baidoun et al., 2021; Shaham et al., 2025). Its insidious onset, pronounced genetic heterogeneity, and relentless propensity for metastasis make it a moving target that repeatedly outmaneuvers conventional treatment, underscoring an urgent and still-unmet need for therapies that are simultaneously more potent, more selective, and far less toxic (Singh, 2025). For more than half a century, 5-fluorouracil (5-FU) has remained the backbone of CRC chemotherapy; yet its clinical value is increasingly constrained by a well-documented therapeutic gap. 5-FU lacks tumor selectivity and distributes indiscriminately to healthy tissues, producing dose-limiting systemic toxicities such as myelosuppression, mucositis, and chemotherapy-induced diarrhea (Alzahrani et al., 2023). More critically, a large proportion of tumors develop intrinsic or acquired resistance through altered drug metabolism, thymidylate synthase overexpression, evasion of apoptosis, and adaptive remodeling of the tumor microenvironment, ultimately driving relapse and mortality in metastatic disease (Barathan et al., 2024; Gmeiner, 2023). This narrow therapeutic window, efficacy perpetually shadowed by toxicity and resistance, defines the central limitation of 5-FU monotherapy (CR-TUM + FU5) and motivates the search for multi-targeted alternatives derived from natural sources and delivered through precision nanocarriers (Fernandez-Munoz, 2025). Among nature’s most compelling anticancer leads is melittin (MLT), the principal 26-amino-acid cationic peptide of honeybee (*Apis mellifera*) venom. Unlike single-target chemotherapeutics, melittin engages tumor cells through a remarkable diversity of mechanisms: it disrupts lipid membranes, triggers mitochondrial reactive oxygen species bursts, and activates the intrinsic apoptotic cascade by upregulating Bax and cytochrome c while suppressing Bcl-2 (Rady et al., 2017; Zhang et al., 2024). In CRC specifically, melittin inhibits tumor growth via endoplasmic reticulum stress and calcium-homeostasis disruption (Luo et al., 2023), and restrains invasion, metastasis, and angiogenesis by downregulating matrix metalloproteinases, epithelial–mesenchymal-transition markers, and vascular endothelial growth factor (Wang, 2024). It further modulates pivotal oncogenic signaling nodes, including the NF-κB and PI3K/Akt pathways, and exerts immunomodulatory effects that reshape the tumor microenvironment (Pandey et al., 2023). These converging actions position melittin peptides as a mechanistically broad and promising candidate for CRC therapy. The clinical translation of melittin has nonetheless been hindered by its non-specific cytolytic and hemolytic activity, rapid plasma degradation, and unfavorable pharmacokinetics (Wang A et al., 2022). Liposomal encapsulation offers an elegant solution to these barriers. As biocompatible phospholipid vesicles, liposomes shield the labile peptide from enzymatic degradation, mask its hemolytic surface activity to curtail off-target toxicity, and prolong systemic circulation (Nsairat et al., 2022). In addition, PEGylated liposomes have been reported to favour accumulation in tumor tissue through the enhanced permeability and retention (EPR) effect, enable controlled and stimulus-responsive drug release, and permit surface functionalization for active tumor targeting (AlSawaftah et al., 2021). Consistent with this rationale, nano-formulated melittin has demonstrated potent antitumor efficacy against colon carcinoma *in vivo* while sparing healthy tissue, confirming that nanocarrier delivery can decouple melittin’s therapeutic potency from its toxicity (Cheng and Xu, 2020; Lv et al., 2021; Yu et al., 2020). Accordingly, liposomal melittin peptides (CR-TUM + LIP-MLT) represent a rational strategy to harness the venom peptide’s multi-mechanistic power safely. To further enhance efficacy and offset the oxidative burden associated with venom-peptide therapy, the present work pairs melittin with chrysin, a naturally occurring 5,7-dihydroxyflavone abundant in honey and plant extracts. Chrysin exhibits antioxidant, anti-inflammatory, and anticancer activities, selectively modulating signaling pathways that govern inflammation, proliferation, angiogenesis, apoptosis, and metastasis (Kasala et al., 2015; Raina et al., 2024). Its clinical utility, however, is limited by poor aqueous solubility and rapid hepatic and gastrointestinal metabolism, which curtail systemic bioavailability (Kurkiewicz et al., 2025). Encapsulation within niosomes, non-ionic surfactant-based nanovesicles, overcomes these constraints by improving solubility, stability, cellular uptake, and controlled release, thereby amplifying chrysin’s anticancer performance in colorectal and other malignancies (Moammeri et al., 2023; Mollazadeh et al., 2024). Niosomal chrysin (CR-TUM + NsCR) thus provides a complementary, antioxidant-rich partner for liposomal melittin. In this context, the present study aimed to investigate the antitumor activity of melittin-loaded liposomes and nanoformulated niosomal chrysin in a DMH-induced CRC rat model. The combination with chrysin nanoparticles, a natural antioxidant, reduces the systemic toxicity associated with the venom peptide and enhances its efficacy against cancer cells. The study specifically focused on downregulating the overexpression of apoptotic and anti-apoptotic genes, cell-cycle-arrest and inflammation-related genes, alongside histopathological and immunohistochemical evaluation. By strategically integrating natural bioactive compounds with nanotechnology, this combined liposomal melittin/niosomal chrysin platform (CR-TUM + LIP-MLT/NsCR) holds significant promise for advancing potent CRC therapies with enhanced delivery that minimize off-target effects and improve treatment outcomes benchmarked against the untreated colorectal tumor (CR-TUM) and conventional 5-FU (CR-TUM + FU5) groups for patients worldwide.

## 2 Materials and methods

### 2.1 Drugs and chemicals

1,2-Dimethylhydrazine (DMH) was obtained from Sigma Aldrich (Molecular Weight: 133.02, CAS Number: 306–37–6, 10 gm), 5-fluorouracil (FU5) was purchased from Sigma-Aldrich (Product No.: 343,922), and chrysin (purity ≥98%, CAS Number: 480–40–0, 5 gm) was purchased from Cayman Chemical (Ann Arbor, MI, United States). Phospholipon 90H (hydrogenated phosphatidylcholine, CAS-Number: 97,281–48–6, 10 gm) was obtained from Lipoid GmbH (Ludwigshafen, Germany. Cholesterol (CAS-Number: 57–88–5, 25 gm), polyethylene glycol 4,000 (CAS-Number:25,322–68–3, PEG_4000_, 2 gm) and Span 60 (sorbitan monostearate, CAS-Number: 215–664–9, 500 gm)) were purchased from Sigma-Aldrich (St. Louis, MA, United States).

### 2.2 Formulation and characterization of PEG liposomes-based melittin peptides

Melittin peptides derived from honeybee (*A. mellifera*) venom were purchased from Sigma-Aldrich (St. Louis, MO, United States) (Product No. M2272, ≥85% purity by HPLC, lyophilized powder, Mw. 2,846.5), a white lyophilized powder stored at −20 °C before use. Blank liposomes were prepared using the ethanol injection method as previously reported (Mostafa et al., 2024). Phospholipon 90H (hydrogenated phosphatidylcholine), cholesterol, and polyethylene glycol 4,000 (PEG_4000_) were accurately weighed at a weight ratio of 13: 3: 1 and dissolved in absolute ethanol by heating at 70 °C with magnetic stirring to ensure complete solubilization of the lipid components. The resulting lipid-ethanol solution was rapidly injected into preheated distilled water (70 °C) maintained under continuous stirring at 500 rpm, leading to spontaneous formation of blank liposomes upon contact with the aqueous phase. Stirring continued for an additional 30 min at 40 °C to facilitate residual ethanol evaporation. The liposomal suspension was then subjected to probe sonication (30 s) for size homogenization. For melittin peptide encapsulation, lyophilized melittin peptides were dissolved in double-distilled water and mixed with a prepared blank liposomal suspension. The mixture was incubated at 37 °C for 4 h with gentle stirring to allow passive loading of venom peptides into the liposomal structures. The prepared melittin peptides-loaded liposomes were characterized for the hydrodynamic diameter and polydispersity index of as determined by dynamic light scattering using a Zetasizer Nano ZS (Malvern Instruments, UK) at 25 °C. The surface charge was evaluated via zeta potential measurements using the same instrument. The samples were diluted 1:100 (v/v) with distilled water prior to analysis. The morphology and structural characteristics of liposomes were examined by scanning electron microscopy (SEM, JSM-6510, JEOL, Tokyo, Japan), as described in Figures 1A–C. Briefly, a drop of liposomal suspension was deposited on a silicon wafer, air-dried, sputter-coated with gold, and imaged at an accelerating voltage of 10 kV.

**FIGURE 1:**
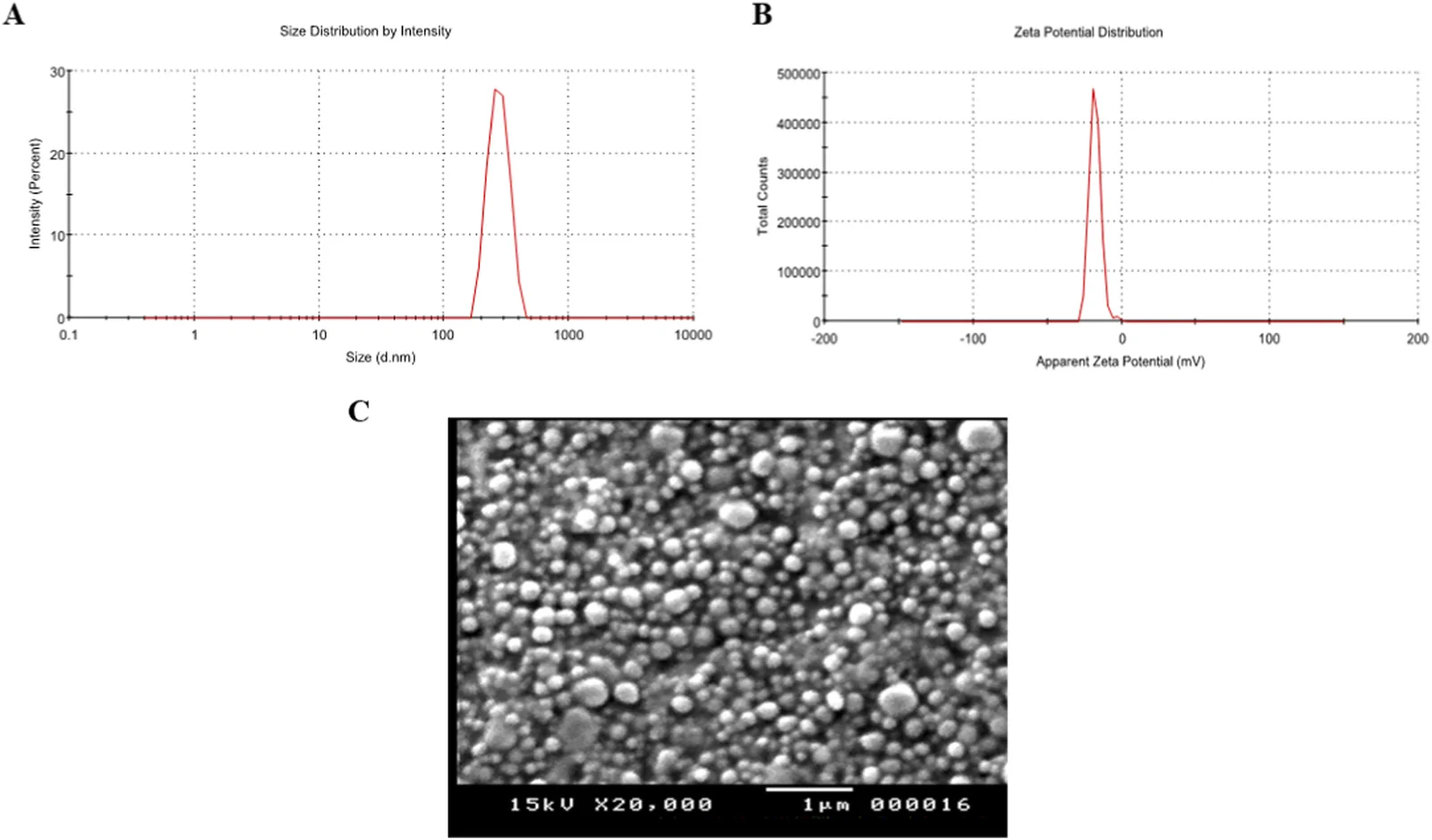
Characterization of bee venom-derived melittin peptides encapsulated liposomal systems zeta potential analysis **(A, B)** scan electron microscope **(C)**.

### 2.3 Preparation and characterization of chrysin-loaded niosomal systems

Chrysin (purity ≥98%, CAS Number) was purchased (5 gm) from Cayman Chemical (Ann Arbor, MI, United States). Chrysin-loaded niosomes were prepared using the ethanol injection method as previously reported (Abd El-Emam et al., 2024). Span 60 (sorbitan monostearate), cholesterol, polyethylene glycol 4,000 (PEG_4000_), and chrysin were accurately weighed at a weight ratio of 19: 7: 1: 4 and dissolved in absolute ethanol under gentle heating at 60 °C with continuous magnetic stirring until complete solubilization of all components was achieved. The resulting homogeneous organic phase was maintained at 60 °C to prevent precipitation. Distilled water was preheated to the same temperature (60 °C) and maintained under vigorous stirring at 500 rpm in a jacketed beaker equipped with a temperature control system. The ethanolic solution containing the niosomal components and chrysin was then rapidly injected into the aqueous phase through a fine syringe needle (27G), leading to the spontaneous self-assembly of chrysin-loaded niosomes upon contact with water because of the reduction in solvent polarity. Stirring continued for 45 min at 50 °C to allow complete evaporation of residual ethanol from the system. The niosomal suspension was subsequently cooled to room temperature under gentle stirring and subjected to bath sonication to reduce the particle size and achieve a more uniform size distribution with improved homogeneity. The hydrodynamic diameter and polydispersity index (PDI) of the chrysin-loaded niosomes were determined by dynamic light scattering using a Zetasizer Nano ZS (Malvern Instruments, UK) at 25 °C. The surface charge was evaluated by zeta potential measurements using the same instrument based on the electrophoretic light scattering technique. Prior to analysis, the samples were appropriately diluted with distilled water (1:100, v/v) to avoid multiple scattering effects. A drop of the niosomal dispersion was placed on a silicon wafer or aluminum stub, air-dried at room temperature, sputter-coated with a thin layer of gold to enhance conductivity, and visualized under scanning electron microscopy (SEM 1400, Jeol, Tokyo, Japan) at an accelerating voltage of 10–15 kV. The encapsulation efficiency of chrysin in the niosomal formulation was determined spectrophotometrically at 267 nm using a UV-visible spectrophotometer (Shimadzu, Kyoto, Japan), as described in Figures 2A–C. Briefly, the niosomal suspension was centrifuged at 15,000 rpm for 1 h at 4 °C to separate free chrysin from the encapsulated drug. The supernatant containing unentrapped chrysin was collected and appropriately diluted, and the absorbance was measured at 267 nm against a blank. The amount of encapsulated chrysin in niosomes (NsCR) was calculated by subtracting the free drug concentration from the total drug initially added. All measurements were performed in triplicate, and the results are expressed as mean ± standard deviation.

**FIGURE 2:**
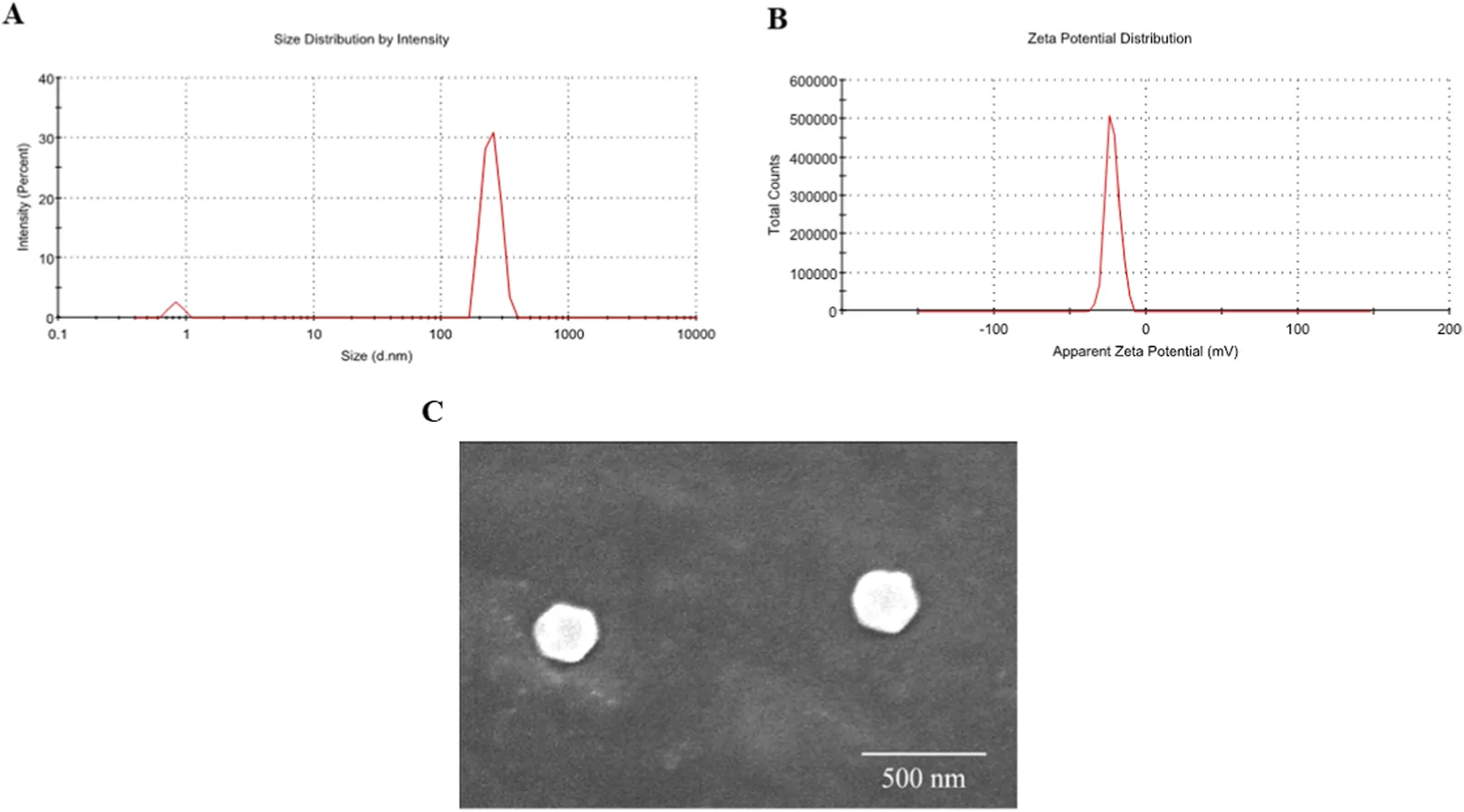
Characterization of chrysin-loaded niosome by pay zeta potential analysis **(A, B)** scan electron microscope **(C)**.

### 2.4 Induction of colorectal tumor models and experimental design

All animal experiments followed the National Institutes of Health guide for the care and use of Laboratory animals (NIH Publications No. 8023, revised 1978). And the study was approved by the IACUC Zagazig Institutional Animal Care and Use Committee (ZU-IACUC/2f/188/2024) of the Faculty of Veterinary Medicine, Zagazig University, Zagazig, Egypt.

One hundred and twenty Sprague-Dawley rats (6–7 weeks old; 150 ± 15 g) were attained from the Laboratory Animals unit, Faculty of Veterinary Medicine, Zagazig University, Egypt, that were housed in a controlled pathogen-free environment and allowed to acclimatization period. Colorectal cancer (CRC) was induced in experimental rats utilizing 1,2-Dimethylhydrazine (DMH). DMH was dissolved in 1 mM EDTA buffer containing 1 mM sodium bicarbonate (pH 6.5) and administered via subcutaneous injection at a dose of 40 mg/kg body weight, twice per week for 8 weeks according to (Ibrahim et al., 2023). Rats were divided randomly into six caged groups each with five replicates (*n* = 20/group). The first group was healthy control group (CON group) and received normal saline. The second group, named the CR-TUM group, was administered DMH (40 mg/kg, S/C for 8 weeks) with no treatment as a colorectal tumor positive control. The third one was CR-TUM + FU5, which was injected DMH for 8 weeks and then treated with 5-Fluorouracil (5-FU, 50 mg/kg/week, intraperitoneal (i.p) according to (Ibrahim, Khater, Abdelfattah-Hassan, Alqahtani, Metwally, Bazeed, Elgamal, Sheraiba, Hussein, Ali Alasmary, Salem, Ali and Mahfouz, 2023). The fourth was injected DMH for 8 weeks and then treated with liposomal honey bee venom derived melittin peptides (CR-TUM + LIP-MLT) at a dose level of (1 mg/kg/day, (i.p) according to (Luo et al., 2023) for 8 weeks. The fifth was injected DMH for 8 weeks and treated with niosomal chrysin (CR-TUM + NsCR) at a dose of 50 mg/kg orally for 8 weeks according to (El-Marasy et al., 2023). The sixth group was injected with DMH for 8 weeks and then treated with the combined therapy comprising LIP-MLT and NsCR (CR-TUM + LIP-MLT/NsCR) at the same doses mentioned above. The two nanoformulations were prepared and administered separately (LIP-MLT intraperitoneally and NsCR orally) and were not mixed before administration. The doses were selected from previously published *in vivo* studies and our previous work with the same DMH model; a formal dose-finding study was not performed. Melittin was used at a low, previously tolerated dose (Luo et al., 2023) because free melittin has a narrow therapeutic window due to its hemolytic and cytolytic activity. The dose of niosomal chrysin (50 mg/kg) was lower than the oral doses of free chrysin (100–200 mg/kg) reported to reduce DMH-induced colorectal lesions in rats (Sequetto et al., 2013), taking into account the improved solubility and bioavailability of chrysin-loaded nanovesicles (El-Marasy, AbouSamra, El-Mosallamy, Emam, Mabrok, Galal, Ahmed-Farid, Abd El-Rahman and Moustafa, 2023). The combination group received the same dose of each agent as the corresponding monotherapy, to allow direct comparison with each single agent.

### 2.5 Evaluation of clinical manifestations associated with colorectal cancer

Body weight was recorded in all rats following the induction of colorectal cancer (CRC). Mortality was recorded throughout the treatment period, and the time to death of each rat was used for survival analysis; rats surviving to the end of the experiment were censored at the final time point, and all episodes of diarrhea were graded throughout the treatment period according to a standardized scale (Arbuckle et al., 2000). Diarrhea severity is graded as follows: Grade 0 indicates absence of diarrhea; Grade 1 (mild) represents an increase of 2–3 stools per day; Grade 2 (moderate), an increase of 4–6 stools per day, nocturnal bowel movements, or moderate abdominal cramping; Grade 3 (severe) is characterized by an increase of 7–9 stools per day, fecal incontinence, or severe cramping; and grade 4 (life-threatening) entails an increase of 10 or more stools per day. The presence of bloody diarrhea or rectal bleeding was also assessed and scored using established procedures (Wang et al., 2017) as follows: 0, negative (no diarrhea), 2 = Hemoccult; and 4, gross bleeding.

### 2.6 Sample collection

At the end of the experimental period, all rats were anesthetized with ketamine hydrochloride (30 mg/kg body weight) and euthanized by cervical dislocation. Blood samples were collected either with EDTA for hematological analysis or without anticoagulants to obtain serum for biochemical and carcinogenesis biomarker analyses. Colon tissues were excised, measured, and washed then divided into three sections: one was stored at −80 °C for molecular inquiries, another was used for histological and immunohistochemical examinations, and the third was homogenized and centrifuged for the assessment of inflammatory and oxidant/antioxidant biomarkers.

### 2.7 Assessment of blood and serum parameters

Red blood cell count and hemoglobin concentration were measured in fresh blood samples as described by (Hewitt, 1984). Alanine Aminotransferase (ALT) and Aspartate Aminotransferase (AST) levels were measured using assay kits (Chema Diagnostica, Monsano, Italy). Urea and creatinine levels were assessed using commercial kits from Biodiagnostic (Dokki, Giza, Egypt**.**

### 2.8 Assessment of circulating biomarkers for colorectal cancer

ELISA kits from MyBiosource (San Diego, CA, United States) were used to measure the levels of serum carcinogenic biomarkers, including carbohydrate antigen (CA19.9), carcinoembryonic antigen (CEA), and colon cancer-specific antigen (CCSA) (MBS7607928, MBS2512373, and MBS726087), respectively, following the manufacturer’s Sandwich-ELISA principle.

### 2.9 Assessment of colonic oxidants/antioxidants biomarkers in CRC induced rats

Colon tissue homogenate was used to estimate malondialdehyde (MDA), a lipid peroxidation marker, and total antioxidant capacity (TAC) using specific kits from MyBioSource (Cat# MBS508035) and (Cat# MBS169313, respectively), following the manufacturer’s instructions. The activities of superoxide dismutase (SOD), catalase (CAT), and glutathione peroxidase (GSH-Px), as well as the reactive oxygen species (ROS) content in colonic tissues, were measured using specific ELISA kits (MyBioSource, San Diego, United States). Nitric oxide (NO) concentration was determined in the colonic homogenate supernatant as described by (Miranda et al., 2001), with a modification involving zinc sulfate for protein precipitation. Absorbance was measured at 540 nm and NO levels were quantified as nmol/g of tissue**.**

### 2.10 Analyzing cytokines via enzyme linked immunosorbent assay (ELISA) in colonic tissues in CRC induced rats

Colon interleukin (IL-1β, Cat #BMS630), (IL-6, Cat #BMS625), (IL-2, Cat #BMS634), (IL-18, Cat # KRC2341), (IL-17A, Cat # BMS635), (IL-10, Cat #BMS629), interferon (IFN-γ, Cat # BMS621), tumor necrosis factor-α (TNF-α; Cat #BMS622), and nuclear factor-kappa B (NF-KB, Cat # EEL138) levels were quantified using commercially available Thermo Fisher ELISA kits, in accordance with the manufacturer’s guidelines.

### 2.11 Quantitative real-time PCR (qRT-PCR) analysis

The transcriptional levels of inflammatory mediators including interleukin 1-beta (*IL-1*β), tumor necrosis factor-α (*TNF*-α), Nuclear Factor-kappa B (*NF*-*κB*), transforming growth factors beta (*TGF*-*β*), cyclooxygenase-2 (*COX*-2), C-C motif chemokine ligand 22 (*CCL22*), chemokine Ligand 8 (C-X-C Motif) (*CXCL8*), chemokine (C-C motif) ligand 9 (*CCL9*), signal Transducer and Activator of Transcription 3 (*STAT3*) and mothers against decapentaplegic homolog 4 (*SMAD4*), innate immune sensing genes including cyclic GMP-AMP synthase (*cGAS*),stimulator of interferon response *cGAMP* interactor 1(*STING* ***),*** TANK-binding kinase 1 (*Tbk1*)interferon regulatory factor 3 (*Irf3*), apoptotic and antiapoptotic mediators including caspase3, caspase 8, *BCL2* associated X (*BAX*), tumor protein p53 (*p53*), cellular FLICE-like inhibitory protein (c-FLIP) (B-cell leukemia/lymphoma 2 protein, (Bcl-2) and survivin and cell cycle arrest regulatory genes including cyclin-dependent kinase 4 (*CDK4*), cyclin-dependent kinase 6 (*CDK6*), krüppel-like factor 4 (*KLF4*), cyclin D1, cyclin-dependent kinase inhibitor 1B (*p27*), cyclin-dependent kinase inhibitor 1A (p21) and β-catenin and mechanistic target of rapamycin (*mTOR*), matrix metalloproteinase-9 (*MMP-9*) and vascular endothelial growth factor (*VEGF*) were estimated in colonic tissues of all experimental groups. Total RNA RNA was isolated from colon tissues using RNeasy Mini kits (Qiagen, CA, United States) following the manufacturer’s protocol. The purity and concentration of the extracted RNA were verified using a NanoDrop ND-1000 Spectrophotometer (Thermo Fisher Scientific Inc., Waltham, MA, United States). A reaction mixture of 25 μL was created by mixing 10 μL of 2× concentrated HERA SYBR Green RT-qPCR Master Mix (Willow Fort, Nottinghamshire, UK), 1 μL of 20× concentrated RT Enzyme Mix, 0.5 μL of each primer (20 pmol), 5 μL of RNase-free water, and 3 μL of RNA template. A one-step real-time PCR system was used for all the experiments. Each PCR reaction was performed using a Stratagene MX3005P real-time PCR system (Stratagene, La Jolla, CA, United States). PCR amplification was conducted over 40 cycles; each cycle composed of a 15-s denaturation at 95 °C followed by a 30-s annealing/extension at 60 °C. Gene expression levels were quantified relative to a reference gene, glyceraldehyde 3-phosphate dehydrogenase (*GAPDH*), using the ^2−ΔΔCT^ method (Livak and Schmittgen, 2001). Specific primer sequences for the tested genes are listed in Table 1.

**TABLE 1:** Primer sequences utilized for rRT-PCR analysis of targeted gene expression.

Target genes	Primer sequence (5′–3′)	Product size	Accession no.
Cell cycle arrest regulatory genes	​	​	​
*CDK4*	F- CAGATAAAGGGCCACCTCCG R- CCATAGGCACCGACACCAAT	110	NM_053593.3
*CDK6*	F- CTCCAGACTTTGGGGAACCG R- AAAAGCAACCGCCCTTACGA	167	XM_006236020.5
*KLF4*	F-GGGCAAGTTTGTGCTGAAGG R-GGGACGGCCTCTTGCTTAAT	171	XM_032903884.1
Cyclin D1	F- CCAAAATGCCAGAGGCGGAT R- GCAAGGAGTCAGATGTGCCC	123	AB042564.1
*P21*	F- TCCCCATCTTCTGCCTTAGC R- GTATATCGAATTGCACGAGGGGA	116	U24174.1
*P27*	F- AGATACGAGTGGCAGGAGGT R- ATGCCGGTCCTCAGAGTTTG	171	D83792.1
β-catenin	F- GACCAGTTCCCTCTTCAGGAC R- CTCACTTAAGCTCGCTCCTGT	107	AF397179.1
Ions change regulatory genes	​	​	​
***CGAS***	F- AAGACTGGCTCGGCACAAAA R- GCGCCATGTCTCTCCTTGAA	107	NM_001444196.1
***STING***	F- GCCATGTCACAGGATGGCAA R- TTGGTAGACAATGAGGCGGC	138	NM_001109122.1
***TBK1***	F- TCGTGAGGAGAAGCGTCTGA R- AGACATTTGCAGTAGCCCCT	100	NM_001106786.1
***IRF3***	F- CAAAACCAGTGCTGACACCC R- CAAAATCAGCGGTTTCGGGG	111	NM_001006969.1
*MMP9*	F- CCCGAGACCTGAAAACCTCC R- GGCCTTTAGTGTCTCGCTGT	188	NM_031055.2
*VEGF*	F- GCCTCACAGGCCTTCTTCAT R- TGAGATAGTACCCTGGCCCC	185	NM_001438359.1
*mTOR*	F- CTGCACTTGTTGTTGCCTCC R- TGGTCTAGCGTGCGAACAAT	164	NM_019906.2
Inflammatory genes	​	​	​
*NF-κB*	F-CAGGACCAGGAACAGTTCGAA R-CCAGGTTCTGGAAGCTATGGAT	150	NM_199267.2
*COX-2*	F-GTGCTTGGCTTGTGACTTTGG R-ACACATTGCATGGGGCCTAC	169	S67722.1
*IL-1*β	F- AGCTTCAGGAAGGCAGTGTC R- ATCCCACGAGTCACAGAGGA	150	NM_031512.2
*TNFα*	F-CAGCCGATTTGCCATTTCA R-AGGGCTCTTGATGGCAGAGA	61	L19123.1
*CCL22*	F- CTCTCGTCCTTCTTGCTGTGG R- GGGTGACGGATGTAGTCCTG	100	NM_057203.1
*CCL9*	F- TAGCAAGACAGAGCCCGAAAC R- GCTAAAAAGCTGATGTCCTTGCT	192	NM_001012357.1
*STAT3*	F- GGAAAGTATTGTCGCCCCGA R- TGGTATTGCTGCAGGTCGTT	115	NM_012747.3
*SMAD4*	F-TTGCATTCCAGCCTCCCATT R-GTTGACCCAAGCAAAAGCGA	177	NM_019275.3
*TGF-β*	F- GACTCTCCACCTGCAAGACC R- GGACTGGCGAGCCTTAGTTT	100	NM_021578.2
Apoptotic and antiapoptotic related genes	​	​	​
Caspase*-3*	F-GCAGCTAACCTCAGAGAGACATTC R-ACGAGTAAGGTCATTTTTATTCCTGACTT	69	NM_012922.3
Caspase*-8*	F- AGAGAAGCAGCCTATGCCAC R- GCCAGTCCGCCAAAGTTTAC	146	NM_022277.2
*P53*	F-CATGAGCGTTGCTCTGATGGT R-GATTTCCTTCCACCCGGATAA	67	NM_030989.3
*Bcl-2*	F- TGTGGAGAGCGTCAACAGG R- CAGAGACAGCCAGGAGAAATCA	178	NM_016993.2
*BAX*	F- ATGGAGCTGCAGAGGATGA R- CCAGTTTGCTAGCAAAGTAG	199	XM_032893644.1
*Survivin*	F- GGAACTGGAAGGCTGGGAAC R- TGGTCTCCTTTGCAATTTTGTTCT	167	AF276775.1
*FLIP*	F- ATCCAGAAGTACACGCAGTCC R- TGATATTCCACACACTGGCTCC	135	NM_057138.3
β-actin	F- GCAAACATCCCCCAAAGTTCT R- ATGCCGTGGATACTTGGAGTG	101	V01217.1
*GAPDH*	F- ATGACTCTACCCACGGCAAG R- AGCATCACCCCATTTGATCTTGA	125	X02231.1

Cyclin-dependent kinase 4 (*CDK4*), cyclin-dependent kinase 6 (*CDK6*), krüppel-like factor 4 (*KLF4*), cyclin D1, cyclin-dependent kinase inhibitor 1B (p27), cyclin-dependent kinase inhibitor 1A (*p21*) and β-catenin, *BCL2* associated X (BAX), tumor protein *p53* (*p53*), cellular FLICE-like inhibitory protein (c-*FLIP*) (B-cell leukemia/lymphoma 2 protein, (*Bcl-2*) and surviving, cyclic GMP-AMP, synthase (*cGAS*),stimulator of interferon response cGAMP, interactor 1(*STING* ***),*** TANK-binding kinase 1 (*Tbk1*)interferon regulatory factor 3 (*Irf3*), mechanistic target of rapamycin (*mTOR*), matrix metalloproteinase-9 (*MMP-9*) and vascular endothelial growth factor (*VEGF*) were estimated in colonic tissues of all experimental CRC, induced groups interleukin beta (*IL-β*), tumor necrosis factor-α (*TNF-*α), B, Nuclear Factor-kappa B (*NF-κB*), transforming growth factors beta (*TGF-β*), cyclooxygenase-2 (*COX-2*), C-C motif chemokine ligand 22 (*CCL22*), chemokine Ligand 8 (*C-X-C Motif*) (*CXCL8*), chemokine (C-C motif) ligand 9 (*CCL9*), signal Transducer and Activator of Transcription 3 (*STAT3*) and mothers against decapentaplegic homolog 4 (*SMAD4*), Glyceraldehyde 3-phosphate dehydrogenase (*GAPDH*).

### 2.12 Histopathological architecture

Colon tissue samples were preserved for histological processing in 10% formal saline. Specimens were cleared in xylene, embedded in paraffin wax, and dehydrated using a series of graded ethanol solutions. Serial sections with a thickness of 5 µm were created using a rotating microtome (Leica®). Hematoxylin and eosin (H&E) staining and DPX mounting were performed on these deparaffinized sections(Suvarna et al. 2018).

### 2.13 Immunohistochemistry

Paraffin sections were stained by immunohistochemistry (IHC) according to (Hsu et al., 1981) using Anti-VEGF Receptor 1 antibody [Y103] (ab32152) at dilution 1/100 and Anti-MMP9 antibody [EP1254] (ab76003) at dilution 1/1000, Abcam, UK, according to the manufacturer’s protocol. The sections were deparaffinized, hydrated, and stained using DAB chromogenic agent (Expose mouse and rabbit specific HRP/DAB detection kit, abcam; Ready–to-use; Cat.#: ab80436)**.**

### 2.14 Statistical analysis

Quantitative data were subjected to statistical analysis using the SPSS® Statistics program (version 22, Inc., United States), random allocation was done for six caged groups each with five replicates. Normality of variance and homogeneity were determined using the Shapiro-Wilk and Levene tests, respectively. The data were evaluated using one-way analysis of variance (ANOVA), and variances among groups were estimated using Tukey’s *post hoc* test. Statistical significance was assessed at *p*-values 0 < 0.05. Experimental graphs were generated using GraphPad Prism software (Version 8, GraphPad Software Inc.). To integrate the ten inflammatory transcripts into interpretable summary measures, a Composite Inflammatory Index (CII) was derived for each group as the arithmetic mean of the relative mRNA fold-changes of the nine pro-inflammatory and chemokine genes (IL-1β, TNF-α, NF-κB, TGF-β, COX-2, CCL22, CXCL8, CCL9, and STAT3); SMAD4 was retained separately as a tumor-suppressor/protective axis. Groups were plotted in a two-dimensional Inflammatory Balance Map (CII versus SMAD4), with bubble size scaled to NF-κB expression as the master inflammatory switch, and quadrant thresholds set at the across-group medians of each axis. Therapeutic Reversal Efficiency was calculated per gene as (CR-TUM − treated)/(CR-TUM − control) × 100 and averaged across the nine pro-inflammatory genes, expressing the percentage of the DMH-induced transcriptional shift that each treatment restored toward the healthy control (0% = no reversal; 100% = full normalization). All underlying gene-expression values were obtained by one-way ANOVA followed by Tukey’s HSD *post hoc* test (p < 0.05), and composite indices are reported as derived point estimates from the group means. For the network representation, each group’s effect was expressed as the log_2_ fold-change of gene expression relative to CR-TUM, and node area was scaled to the absolute log_2_ fold-change. Gene–gene co-regulation was quantified as the Pearson correlation coefficient of expression across the experimental group means; gene pairs with |r| ≥ 0.9 were connected by an edge, and the two network modules were defined by the sign of each gene’s correlation with an independent treatment-efficacy axis derived from the tumor-burden markers CEA, CA19-9, and CCSA. Therapeutic effectiveness was quantified as the concordance between each treatment’s network-reversal profile—the vector of gene-wise log_2_ fold-changes relative to CR-TUM—and that of the healthy control, computed as the cosine similarity of the two vectors and expressed as a percentage (healthy control = 100%). Network construction, correlation, and concordance analyses were performed in Python (NumPy/Matplotlib). Survival was analyzed using the Kaplan-Meier method, and survival curves were compared using the log-rank (Mantel–Cox) test; hazard ratios (HRs) with 95% confidence intervals (CIs) were estimated by the Mantel–Haenszel method. The number of rats at risk at each week is shown beneath each panel. NR, not reached (survival remained above 50% at week 8).

## 3 Results

### 3.1 Assessment of LIP-MLT/NsCR therapeutic effectiveness in mitigating clinical symptom intensity and serum parameters post CRC induction

The dumbbell representation of the week 1 → week 8 trajectories clearly delineated the divergent clinical course of the experimental groups (Figures 3A–D). With respect to body weight (Figure 3A), the healthy control (CON) group displayed a steady anabolic gain over the study period (308.6 → 340.3 g; +31.7 g), whereas the untreated DMH group (CR-TUM) underwent a pronounced cachexic decline (310.8 → 245.5 g; −65.3 g), consistent with progressive tumor-associated wasting. The conventional 5-fluorouracil group (CR-TUM + FU5) only partially limited this loss (308.9 → 287.6 g; −21.3 g). In contrast, both monotherapies preserved body weight close to baseline (CR-TUM + LIP-MLT, +2.1 g; CR-TUM + NsCR, −4.1 g), while the combined regimen (CR-TUM + LIP-MLT/NsCR) was the only treated group to register a net gain comparable in direction to the control (310.9 → 315.1 g; +4.2 g).

**FIGURE 3:**
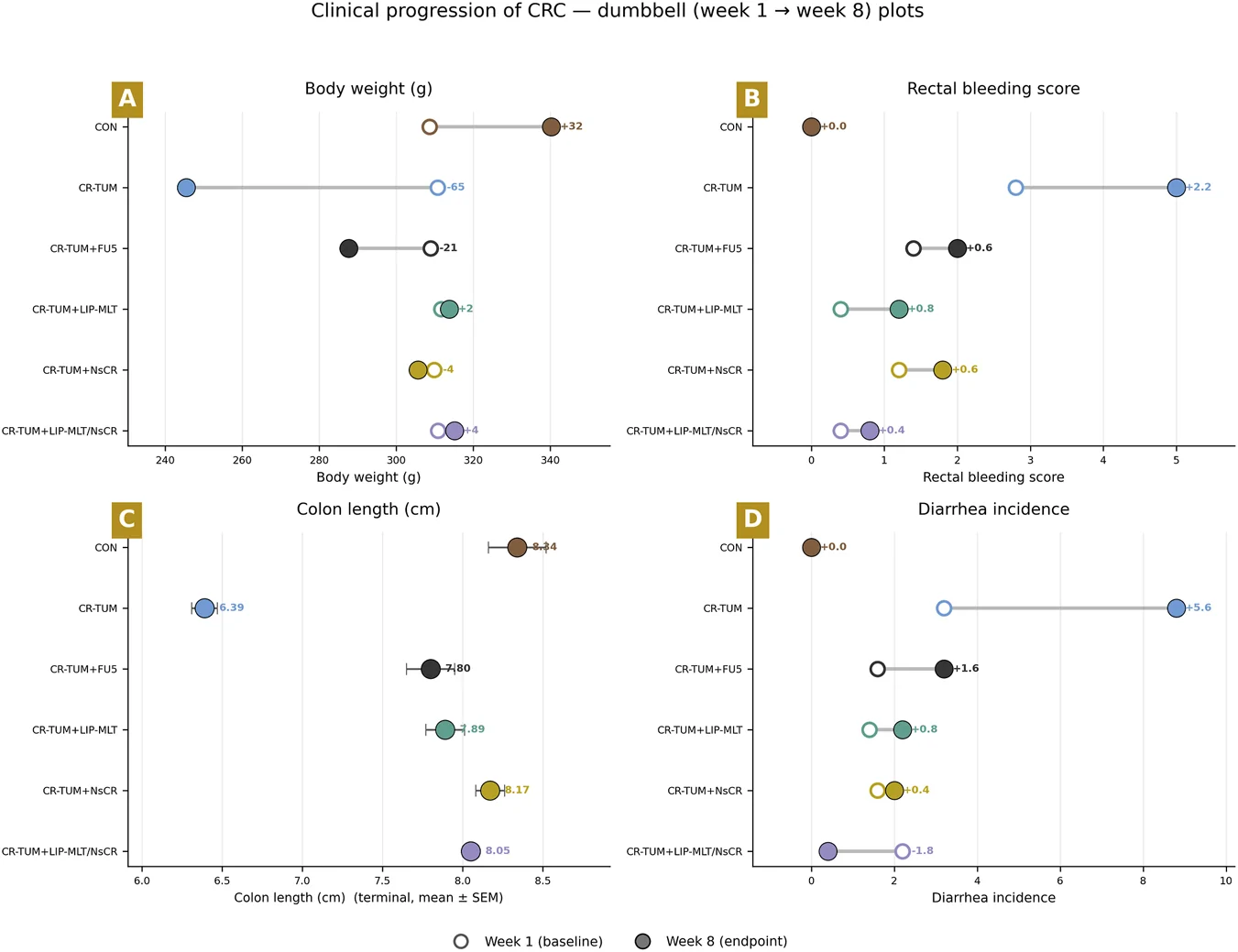
Robust improvement in the clinical progression of colorectal cancer (CRC) signs by nanoengineered liposomal melittin peptides and niosomal chrysin (LIP-MLT/NsCR). Body weight gain **(A)**. Rectal bleeding scoring **(B)**. Dolon length **(C)**. Diarrhea incidence **(D)**. Control (CON): healthy non-CRC group (rats treated with saline). CRC groups: **CR-TUM** (rats injected with 1, 2-dimethylhydrazine [DMH] and left untreated); **CR-**TUM + FU5 (DMH-induced rats treated with 5-fluorouracil, 50 mg/kg/week, intraperitoneal [i.p.], for 8 weeks); CR-TUM + LIP-MLT (DMH-induced rats treated with nanoformulated liposomal melittin 1mg/kgBW/day/ i. p, for 8 weeks); CR-TUM + NsCR (DMH-induced rats treated with nanoformulated niosomal chrysin, 50 mg/kg, orally, for 8 weeks); and CR-TUM + LIP-MLT/NsCR (DMH-induced rats treated with liposomal melittin, 1 mg/kg BW/day/ i. p, plus niosomal chrysin, 50 mg/kg, orally, for 8 weeks as combined therapy. Values are expressed as mean ± SE; means with different letters (a, b, c) differed significantly among groups (p < 0.05).

A parallel pattern was evident for rectal bleeding score (Figure 3B), which remained absent throughout in the CON group and rose sharply in the CR-TUM group (2.8 → 5.0; +2.2). All treatments attenuated this progression, with the smallest terminal score and the flattest trajectory recorded in the CR-TUM + LIP-MLT/NsCR group (0.4 → 0.8; +0.4), followed by the LIP-MLT and NsCR monotherapies, whereas the 5-FU group retained a higher endpoint score (→2.0). Terminal colon length (Figure 3C) was markedly shortened in the untreated CR-TUM group (6.39 cm) relative to the CON group (8.34 cm), reflecting tumor-driven contraction; treatment restored colon length in a graded manner, with the NsCR (8.17 cm) and combined LIP-MLT/NsCR (8.05 cm) groups most closely approximating the control value, exceeding the recovery achieved with 5-FU (7.80 cm).

The diarrhea incidence trajectories (Figure 3D) reinforced these findings: the CR-TUM group escalated steeply across the study (3.2 → 8.8; +5.6), and the 5-FU group showed a sustained increase (1.6 → 3.2; +1.6) in keeping with the known enterotoxicity of 5-fluorouracil. Notably, the CR-TUM + LIP-MLT/NsCR group was the only group to reverse its trajectory, with diarrhea scores declining over the treatment window (2.2 → 0.4; −1.8). Collectively, the dumbbell profiles demonstrate that combined LIP-MLT/NsCR therapy produced the most favorable shift across all four clinical indices, outperforming either monotherapy or the standard 5-FU treatment (p < 0.05). This group also showed marked anemia, as evidenced by the low RBC counts and Hb levels (Table 2). Liver function was severely distributed, as evident from the sharp elevation of liver enzymes, ALT, and AST (increased to 6.6, 2.0-fold respectively vs. compared to the control group). Additionally, the urea and creatinine levels were significantly elevated (*p* < 0.05), reflecting kidney impairment. Regarding tumor markers, the CRC-induced group showed a pronounced increase in CEA (5.3-fold), CA19-9 (7.2-fold), and CCSA (3.0-fold) levels compared to the control, confirming successful disease induction. Kaplan-Meier survival curves are shown in Figure 4. All treatments significantly improved survival compared with untreated CR-TUM rats, with the combined LIP-MLT/NsCR regimen showing the highest survival and lowest risk of death among the treated groups. All groups started with 20 rats, and no deaths occurred in the CON group. By week 8, the number of rats at risk had declined to 9 in the CR-TUM group, compared with 13, 16, 14, and 17 in the CR-TUM + FU5, CR-TUM + LIP-MLT, CR-TUM + NsCR, and CR-TUM + LIP-MLT/NsCR groups, respectively. Untreated CR-TUM rats showed the lowest survival at week 8 (45.0%), with a median survival of 7 weeks, and differed significantly from the CON group (100%; log-rank p = 0.0001). Median survival was not reached in any treated group, because survival remained above 50% at week 8. Relative to CR-TUM, the combined LIP-MLT/NsCR regimen produced the highest survival (85.0%) and the greatest reduction in the risk of death (HR = 0.20; 95% CI, 0.07–0.61; p = 0.0045), followed by LIP-MLT (80.0%; HR = 0.33; 95% CI, 0.12–0.95; p = 0.039). The improvements with NsCR (70.0%; HR = 0.48; 95% CI, 0.18–1.29; p = 0.147) and 5-FU (65.0%; HR = 0.52; 95% CI, 0.20–1.39; p = 0.193) did not reach statistical significance. nAll groups started with 20 rats at risk, and the number at risk remained unchanged in the CON group throughout the study. By week 8, the number at risk had declined to 9 in the CR-TUM group, compared with 13, 16, 14, and 17 in the CR-TUM + FU5, CR-TUM + LIP-MLT, CR-TUM + NsCR, and CR-TUM + LIP-MLT/NsCR groups, respectively, with the combination group retaining the highest number of rats at risk among the treated groups.

**TABLE 2:** Robust therapeutic effectiveness of nanoengineered liposomal melittin peptides and niosomal chrysin (LIP-MLT/NsCR) on hepatic and renal functions and hematological profiles in colorectal cancer induced rats’ model.

Parameter	Experimental groups							
	CON	CR-TUM	CR-TUM + FU5	CR-TUM+LIP-MLT	CR-TUM + NsCR	CR-TUM+LIP-MLT/ NsCR	SEM	*p*-value
ALT (U/L)	38.28^days^	114.18^a^	116.77^a^	87.81^b^	100.00^b^	68.33^c^	3.06	<0.001
AST (U/L)	58.87^c^	149.76^a^	128.04^a^	76.82^b^	89.00^b^	69.33^b^	3.71	<0.001
Urea (mg/dL)	28.82^b^	46.54^a^	44.47^a^	32.83^b^	35.82^b^	32.18^b^	1.41	<0.001
Creatinine (mg/dL)	0.56^b^	1.12^a^	1.06^a^	0.62^b^	0.66^b^	0.61^b^	0.02	<0.001
RBC (x10^6^/μL)	11.99^a^	8.63^c^	9.37^b^	9.98^b^	10.97^a^	11.88^a^	0.26	0.03
Hb (g/dL)	13.43^a^	8.88^c^	8.83^c^	10.87^b^	11.67^b^	12.17^a^	0.28	0.01
CEA (ng/mL)	0.85^c^	4.78^a^	1.51^b^	2.17^b^	2.41^b^	1.57^b^	0.21	0.02
CA19-9 (U/mL)	0.74^days^	7.60^a^	1.75^c^	2.87^b^	3.54^b^	2.15^c^	0.20	0.03
CCSA (ng/mL)	16.87^days^	67.20^a^	30.83^c^	37.57^b^	39.91^b^	31.82^c^	0.87	<0.001

ALT: alanine transaminase, AST: aspartate transaminase, RBCs: red blood cells, Hb: hemoglobin, Human carcino embryonic antigen (CEA), carbohydrate antigen (CA19-9), colon cancer-specific antigen (CCSA). CON: healthy Non-CRC, group (rats were orally gavaged with saline), CR-TUM, groups including: CRC (rats were injected with dimethylhydrazine (DMH)) and received no treatment; CR-TUM + FU5 (rats were administered DMH, to induce colorectal cancer and subsequently treated with 5-Fluorouracil at a dose of 50 mg/kg/week via intraperitoneal injection for 8 weeks), CR-TUM + LIP-MLT (rats were injected with DMH, and received and treated with nanoformulated liposomal melittin peptides (1 mg/kg BW/day/i. p), CR-TUM + NsCR (rats were injected with DMH, and received nanoformulated niosomal chrysin at the level of 50 mg/kg, orally for 8 weeks), CR-TUM + LIP-MLT/NsCR (rats were injected with DMH, and received nanoformulated liposomal melittin peptides (1 mg/kg BW/day/i.p) and niosomal chrysin at the level of 50 mg/kg, orally for 8 weeks) as combined therapy. Values are means with SEM., Means within a row not sharing a common superscript letter differ significantly (Tukey’s HSD, p < 0.05); a denotes the highest mean.

**FIGURE 4:**
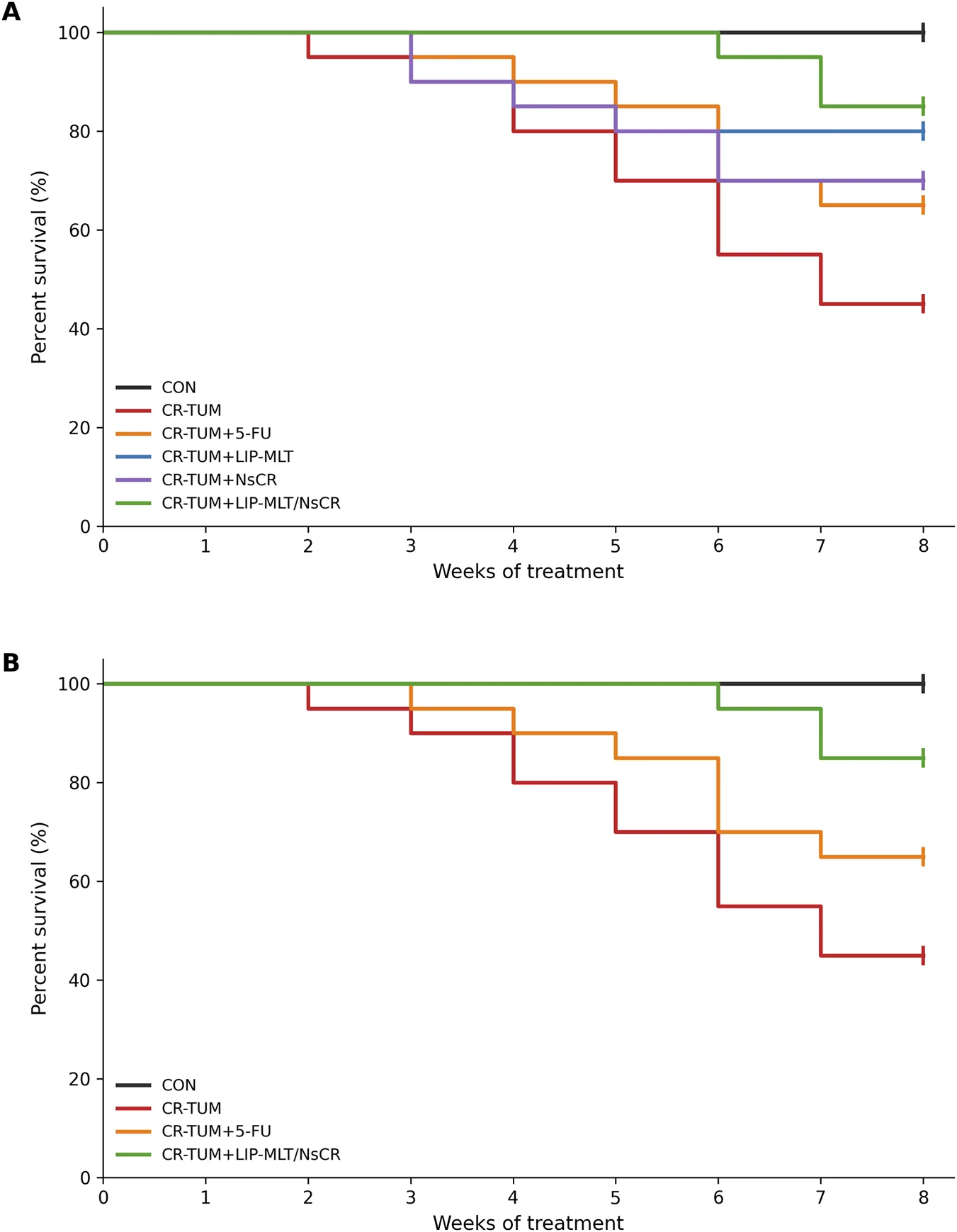
Robust improvement in survivability by nanoengineered liposomal melittin peptides and niosomal chrysin (LIP-MLT/NsCR). Survival percentage across CRC-induced groups **(A)**; survival percentage of non-CRC and DMH-induced CRC groups, comparing untreated, conventional 5-FU, or combined LIP-MLT/NsCR treatment **(B)**. Control (CON): healthy non-CRC group (rats treated with saline). CRC groups: CR-TUM (rats injected with 1,2-dimethylhydrazine [DMH] and left untreated); CR-TUM + FU5 (DMH-induced rats treated with 5-fluorouracil, 50 mg/kg/week, intraperitoneal [i.p.], for 8 weeks); CR-TUM + LIP-MLT (DMH-induced rats treated with nanoformulated liposomal melittin, 1mg/kgBW/day/ i. p., for 8 weeks); CR-TUM + NsCR (DMH-induced rats treated with nanoformulated niosomal chrysin, 50 mg/kg, orally, for 8 weeks); and CR-TUM + LIP-MLT/NsCR (DMH-induced rats treated with liposomal melittin, 1mg/kgBW/day/ i.p., plus niosomal chrysin, 50 mg/kg, orally, for 8 weeks) as combined therapy.

### 3.2 Assessment of oxidative stress and antioxidant defense metrics

Table 3 clearly revealed that, compared with the control group, the untreated **CR-TUM** group exhibited a highly significant increase in oxidative-damage indicators, including MDA (3.5-fold), NO (1.6-fold), and ROS (5.0-fold) versus control. At the same time, the **CR-TUM** group showed a notable decrease in antioxidant enzyme activity, including SOD, CAT, GPx, and TAC, leaving the colonic tissue highly exposed to oxidative injury. In contrast, **LIP-MLT (CR-TUM + LIP-MLT)** and NsCR (CR-TUM + NsCR) monotherapy partially restored these antioxidant defences. Notably, CAT and GPx activities increased significantly with **NsCR** monotherapy, which also markedly lowered the oxidative-stress indicators MDA, NO, and ROS, demonstrating its pronounced efficacy. The combined **LIP-MLT/NsCR** therapy (CR-TUM + LIP-MLT/NsCR) produced the strongest effect against CRC-induced oxidative stress, consistent with a synergistic action that both reinforces endogenous antioxidant mechanisms and scavenges free radicals. By comparison, **5-FU** treatment (CR-TUM + FU5) achieved only moderate reduction, with weaker restoration of SOD, CAT, and GPx and higher residual MDA and ROS than the **LIP-MLT/NsCR** combination, indicating that its primary mode of action is cytotoxic rather than antioxidant.

**TABLE 3:** Robust therapeutic effectiveness of nanoengineered liposomal melittin peptides and niosomal chrysin (LIP-MLT/NsCR) against oxidative stress in colorectal cancer induced rats’ model.

​	Experimental groups							
Parameter	CON	CR-TUM	CR-TUM + FU5	CR-TUM+LIP-MLT	CR-TUM + NsCR	CR-TUM+LIP-MLT/ NsCR	SEM	*p*-value
MDA (nmol/g tissue)	4.11^e^	26.74^a^	20.58^b^	18.55^c^	17.80^c^	15.10^days^	0.32	<0.001
NO (nmol/g tissue)	67.65^days^	230.29^a^	151.84^b^	116.53^c^	121.79^c^	102.81^c^	4.11	0.02
TAC (mol trolox equivalent tissue)	31.99^b^	11.29^e^	18.55^days^	19.68^days^	24.25^c^	40.65^a^	0.81	0.01
SOD (U/mg)	7.22^a^	2.62^b^	3.75^b^	3.89^b^	7.68^a^	9.10^a^	0.35	<0.001
Catalase (U/mg)	7.49^a^	2.63^c^	3.98^b^	4.38^b^	9.10^a^	8.97^a^	0.32	<0.001
GSH-px (U/mg)	10.11^c^	2.45^e^	4.03^days^	4.85^days^	12.31^b^	14.73^a^	0.37	0.03
ROS (µmol/g tissue	42.03^e^	223.01^a^	192.48^b^	135.01^c^	121.43^c^	101.81^day^	3.74	<0.001

Malondialdehyde (MDA), superoxide dismutase (SOD), glutathione peroxidase (GSH-Px), nitric oxide (NO), reactive oxygen (ROS), and total antioxidant capacity (TAC). CON: healthy Non-CRC, group (rats were orally gavaged with saline), CR-TUM, groups including: CRC (rats were injected with dimethylhydrazine (DMH)) and received no treatment; CR-TUM + FU5 (rats were administered DMH, to induce colorectal cancer and subsequently treated with 5-Fluorouracil at a dose of 50 mg/kg/week via intraperitoneal injection for 8 weeks), CR-TUM + LIP-MLT (rats were injected with DMH, and received nanoformulated niosomal chrysin at the level of 50 mg/kg, orally for 8 weeks), CR-TUM + LIP-MLT/NsCR (rats were injected with DMH, and received nanoformulated liposomal melittin peptides (1 mg/kg BW/day/i.p) and niosomal chrysin at the level of 50 mg/kg, orally for 8 weeks) as combined therapy. Values are means with SEM., Means within a row not sharing a common superscript letter differ significantly (Tukey’s HSD, p < 0.05); a denotes the highest mean.

### 3.3 Quantification of inflammatory and anti-inflammatory cytokines in colonic tissues

The levels of critical pro-inflammatory cytokines, including IL-1β, IL-6, TNF-α, IFN-γ, and NF-κB, were significantly elevated in the untreated **CR-TUM** group (Table 4), consistent with the persistent, elevated inflammation that characterizes cancer development. Conversely, the anti-inflammatory cytokine IL-10 and the protective cytokines IL-2 and IL-17 were significantly (p < 0.05) reduced in the **CR-TUM** group, indicating an immunosuppressive imbalance that favours tumour growth. All treatments markedly reduced the pro-inflammatory markers (NF-κB, TNF-α, IFN-γ, IL-1β, and IL-6), with the combined **LIP-MLT/NsCR** therapy (CR-TUM + LIP-MLT/NsCR) being the most effective at dampening the inflammatory response following CRC induction. In the **5-FU** group (CR-TUM + FU5), pro-inflammatory cytokines were successfully decreased, yet the protective cytokines (IL-10, IL-2, and IL-17) were not restored, remaining low and statistically comparable to those in the **CR-TUM** group. This implies that, although 5-FU is cytotoxic, it lacks the immunomodulatory properties of the **LIP-MLT/NsCR** combination.

**TABLE 4:** Robust therapeutic effectiveness of nanoengineered liposomal melittin peptides and niosomal chrysin (LIP-MLT/NsCR) against inflammatory response in colorectal cancer induced rats’ model.

Parameter	Experimental groups							
	CON	CR-TUM	CR-TUM + FU5	CR-TUM+LIP-MLT	CR-TUM + NsCR	CR-TUM+LIP-MLT/ NsCR	SEM	p-value
IL-1β (pg/mL)	109.70e	264.56a	175.78b	153.26c	128.06d	109.08e	3.31	0.01
IL-6 (pg/mL)	72.30d	296.95a	161.50c	189.19b	207.26b	143.42c	4.60	0.03
IL-8 (pg/mL)	48.22b	301.39a	174.97a	181.74a	151.58b	110.32b	15.91	<0.001
IL-2 (pg/mL)	135.55a	95.94d	99.87c	108.32c	114.31b	122.81b	1.63	<0.001
IL-10 (pg/mL)	132.63b	95.93d	113.36c	122.82b	181.23a	190.14a	2.92	0.02
IL-17A (pg/mL)	45.43c	16.34e	22.58d	26.80d	58.30b	70.44a	1.10	<0.001
IFN-γ (pg/mL)	105.22f	408.63a	333.66b	298.23c	274.57d	203.06e	3.88	<0.001
TNF-α (pg/mL)	85.41d	179.65a	143.11b	146.13b	124.46c	108.92c	3.37	0.03
NF-κB (pg/mL)	30.94e	182.55a	100.19c	103.67c	128.55b	79.67d	2.41	<0.001

Interferon gamma (IFNγ), tumor necrosis factor alpha (TNF-α), and interleukin (IL) Nuclear Factor-kappa B (NF-κB). CON: healthy Non-CRC, group (rats were orally gavaged with saline), CR-TUM, groups including: CRC (rats were injected with dimethylhydrazine (DMH)) and received no treatment; CR-TUM + FU5 (rats were administered DMH, to induce colorectal cancer and subsequently treated with 5-Fluorouracil at a dose of 50 mg/kg/week via intraperitoneal injection for 8 weeks), CR-TUM + LIP-MLT (rats were injected with DMH, and received nanoformulated niosomal chrysin at the level of 50 mg/kg, orally for 8 weeks), CR-TUM + LIP-MLT/NsCR (rats were injected with DMH, and received nanoformulated liposomal melittin peptides (1 mg/kg BW/day/i.p) and niosomal chrysin at the level of 50 mg/kg, orally for 8 weeks) as combined therapy. Values are means with SEM., Means within a row not sharing a common superscript letter differ significantly (Tukey’s HSD, p < 0.05); a denotes the highest mean.

### 3.4 LIP-MLT/NsCR co-therapy dismantles the expression of pro-inflammatory chemokine network in DMH-induced colorectal cancer

The relative mRNA expression of the ten-genes inflammatory panel is summarized as a heatmap in Figure 5A, with all values normalized to the healthy control (CON = 1.0). Induction of colorectal cancer triggered a coordinated pro-inflammatory transcriptional program: relative to CON, the untreated tumour (CR-TUM) markedly upregulated IL-1β (9.0-fold), TNF-α (9.8-fold), *NF-κ*B (9.5-fold), *COX*-2 (9.8-fold), *TGF*-*β* (5.7-fold), *CCL22* (6.0-fold), CXCL8 (7.8-fold), *CCL9* (8.2-fold), and *STAT3* (7.7-fold), each occupying the highest Tukey grouping (letter a). In parallel, the tumour-suppressor/protective mediator SMAD4 remained comparatively low in the CR-TUM group (2.5-fold), consistent with an unrestrained, immunosuppressive inflammatory microenvironment. All treatments significantly reversed this signature relative to CR-TUM (asterisks; p < 0.05), but to differing extents. Conventional 5-FU (CR-TUM + FU5) and niosomal chrysin monotherapy (CR-TUM + NsCR) achieved intermediate suppression, with NsCR notably retaining higher *TNF-*α (5.6-fold) and *COX*-2 (5.8-fold) than the melittin-containing arms. Liposomal melittin monotherapy (CR-TUM + LIP-MLT) produced a stronger reduction across most transcripts, whereas the combined LIP-MLT/NsCR regimen was consistently the most effective, returning the pro-inflammatory genes closest to control values: *IL-1*β (3.0-fold), *NF-κB* (3.0-fold), *COX*-2 (2.2-fold), *TGF*-β (2.3-fold), *CCL22* (1.8-fold), *CXCL8* (4.7-fold), *CCL9* (3.1-fold), *STAT3* (2.8-fold), and *TNF*-α (4.6-fold; statistically comparable to LIP-MLT, group c). Expressed relative to the untreated tumor, the combination lowered *COX*-2 4.5-fold, *CCL22* 3.3-fold, *NF-κB* 3.2-fold, and *IL-1*β 3.0-fold versus CR-TUM. Conversely, *SMAD4* was progressively restored by treatment and reached its highest expression in the combination group (7.2-fold; ∼2.9-fold higher than CR-TUM), closely followed by LIP-MLT monotherapy (7.0-fold). The heatmap demonstrates that DMH-induced colorectal tumors mount a strong, coordinated pro-inflammatory and pro-metastatic chemokine response with concurrent suppression of protective *SMAD4* signaling, and that combined LIP-MLT/NsCR therapy restores this inflammatory balance more completely than either monotherapy or conventional 5-FU (p < 0.05). To capture the coordinated behavior of the inflammatory network rather than gene-by-gene changes, the ten transcripts were condensed into a composite inflammatory index and mapped against the protective *SMAD4* axis (Figures 5–B). The untreated CR-TUM group occupied the pro-tumor quadrant, combining the highest inflammatory burden (CII = 8.17, an ∼8-fold over control elevation) with a comparatively low *SMAD4* signal, consistent with an unrestrained, immunosuppressive inflammatory microenvironment. In contrast, the healthy control clustered at baseline (CII = 1.0). All treatments displaced the tumor phenotype leftward toward resolution, but to different extents: 5-FU (CII = 4.99) and NsCR (CII = 4.52) achieved intermediate positions, whereas LIP-MLT (CII = 3.76) and, most markedly, the combined LIP-MLT/NsCR regimen (CII = 3.06) relocated into the resolved/protective quadrant, pairing the lowest inflammatory burden with the highest *SMAD4* expression. The dotted trajectory from CR-TUM to the combination group illustrates this coordinated normalization. The therapeutic reversal efficiency analysis (Figures 5–C) quantified these shifts: the combined LIP-MLT/NsCR therapy reversed 71% of the DMH-induced inflammatory signature toward healthy control, exceeding LIP-MLT (60%), NsCR (50%), and 5-FU (44%) monotherapies.

**FIGURE 5:**
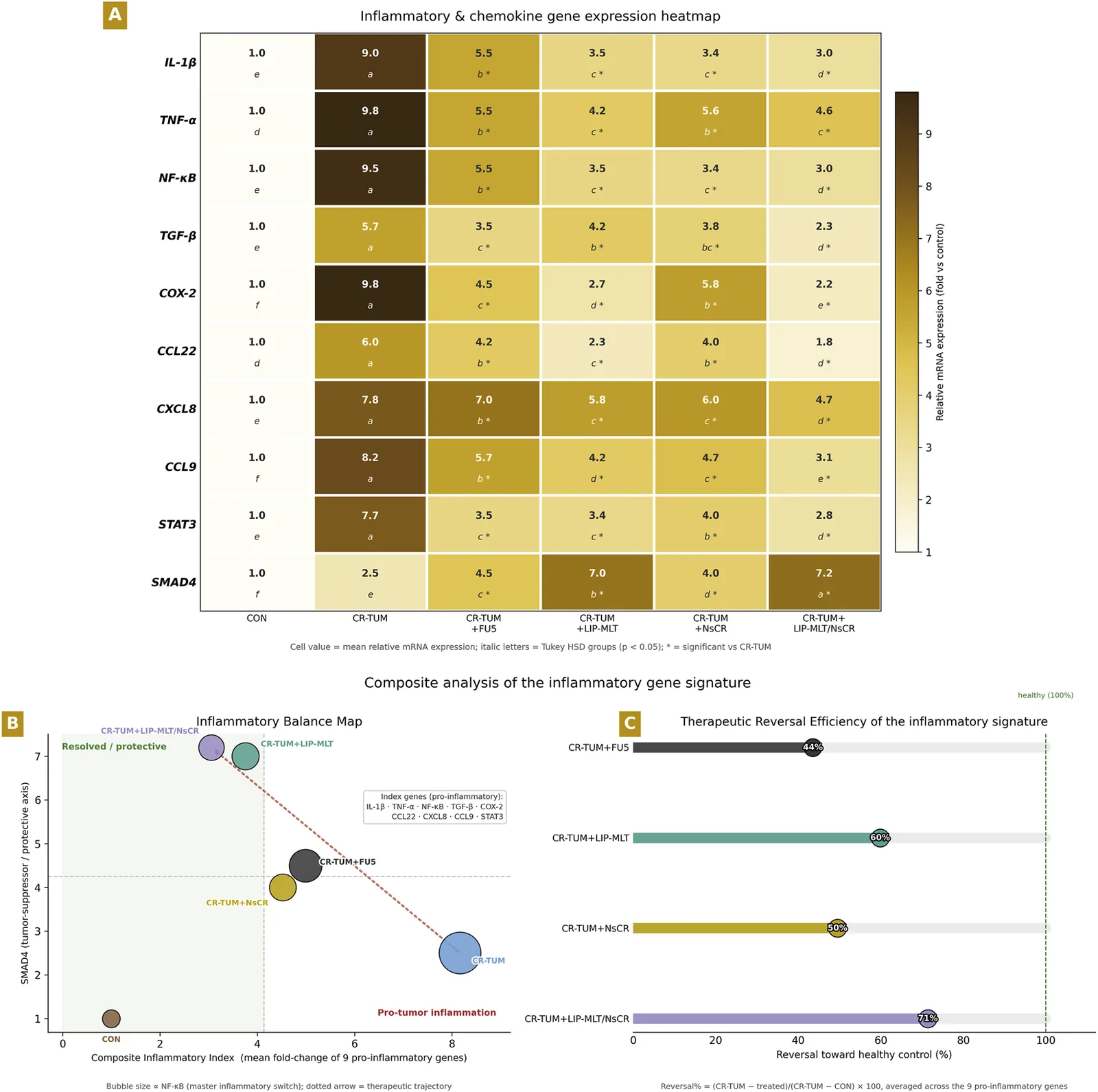
Robust modulation of inflammatory gene expression by nanoengineered liposomal melittin and niosomal chrysin (LIP-MLT/NsCR). **(A)** Heatmap of relative mRNA expression (fold vs. control) for IL-1β, TNF-α, NF-κB, TGF-β, COX-2, CCL22, CXCL8, CCL9, STAT3, and SMAD4 across groups; color intensity reflects expression, cell values are group means, italic letters denote Tukey groupings, and * indicates significance vs. CR-TUM. **(B)** Inflammatory Balance Map plotting each group by its Composite Inflammatory Index (mean fold-change of the nine pro-inflammatory genes) versus SMAD4 (protective axis), with bubble size ∝ NF-κB; the dotted arrow marks the therapeutic trajectory from CR-TUM toward the combination. **(C)** Therapeutic Reversal Efficiency, the percentage of the DMH-induced inflammatory shift normalized toward control [(CR-TUM − treated)/(CR-TUM − control) × 100]. Groups: CON (healthy, saline); CR-TUM (DMH, untreated); CR-TUM + FU5 (5-FU, 50 mg/kg/week, i. p.); CR-TUM + LIP-MLT (liposomal melittin, 1 mg/kg BW/day/i. p.); CR-TUM + NsCR (niosomal chrysin, 50 mg/kg, oral); CR-TUM + LIP-MLT/NsCR (combined), all for 8 weeks. Data are mean ± SE (n = 3/group); one-way ANOVA with Tukey’s HSD, p < 0.05.

### 3.5 Restoring cell-cycle control: LIP-MLT/NsCR suppresses cyclin–CDK drivers and reactivates CDK inhibitors in colorectal cancer

The relative mRNA expression of seven cell-cycle-arrest regulators is presented as polar-area (Nightingale rose) plots in Figures 6A–G, normalized to the healthy control (CON = 1.0). Induction of colorectal cancer produced a hallmark loss of G1/S checkpoint control in the untreated tumour (CR-TUM): the proliferative drivers *CDK4* (8.5-fold), *CDK6* (9.0-fold), cyclin D1 (14.8-fold), and the Wnt effector β-catenin (7.5-fold) were markedly upregulated relative to control (letter a), whereas the CDK inhibitors and tumor-suppressor genes were concomitantly repressed KLF*4* (0.5-fold), *p27* (0.4-fold), and *p21* (0.8-fold) reflecting unchecked cell-cycle progression. Treatment reversed both arms of this dysregulation, and the combined LIP-MLT/NsCR regimen was the most effective across the panel. It produced the greatest suppression of the proliferative drivers, reducing *CDK4* to 3.8-fold, *CDK6* to 3.5-fold, and cyclin D1 to 2.0-fold—corresponding to 2.2-, 2.6-, and 7.4-fold reductions, respectively, relative to CR-TUM—each reaching the lowest Tukey grouping among the treated groups (letter d; p < 0.05). β-catenin was likewise strongly downregulated by the combination (3.0-fold), comparable to liposomal melittin monotherapy, which achieved the single lowest value (CR-TUM + LIP-MLT, 2.6-fold). In parallel, the combination most robustly restored the growth-suppressive machinery, driving *KLF4* (5.1-fold), *p27* (8.7-fold), and *p21* (7.7-fold) to their highest expression among all groups (letter a; p < 0.05), closely followed by LIP-MLT monotherapy (*KLF4;* 4.7-, *p27;* 8.0-, *p21;* 7.5-fold). Conventional 5-FU (CR-TUM + FU5) and niosomal chrysin monotherapy (CR-TUM + NsCR) exerted intermediate effects, with NsCR retaining comparatively higher *CDK4* and *CDK6* (5.0-fold each) and cyclin D1 (3.8-fold) than the melittin-containing arms. Collectively, these polar-area profiles demonstrate that DMH-induced colorectal tumours sustain proliferation through coordinated cyclin–CDK/β-catenin activation and CDK-inhibitor loss, and that combined LIP-MLT/NsCR therapy re-establishes G1/S checkpoint control more completely than either monotherapy or conventional 5-FU (p < 0.05).

**FIGURE 6:**
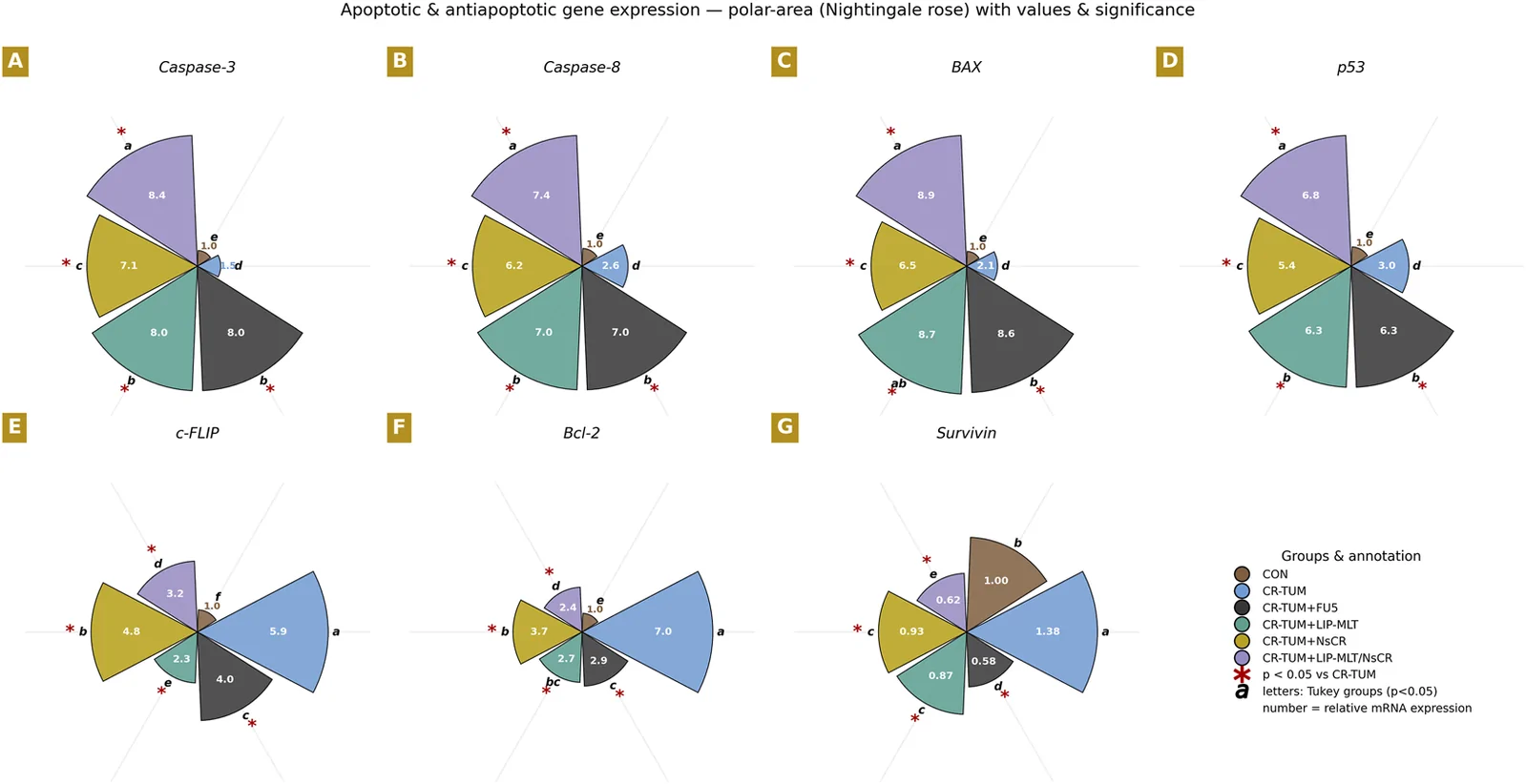
Robust modulation of cell-cycle-arrest regulator gene-expression patterns by nanoengineered liposomal melittin and niosomal chrysin (LIP-MLT/NsCR), presented as polar-area (Nightingale rose) plots. In each panel, every experimental group is drawn as a filled petal whose radius is proportional to the relative mRNA expression (the value is printed on each petal): cyclin-dependent kinase 4 (CDK4, **(A)**, cyclin-dependent kinase 6 (CDK6, **(B)**, Krüppel-like factor 4 (KLF4, **(C)**, cyclin D1 (Cyclin D1, D), cyclin-dependent kinase inhibitor 1B (p27, **(E)**, cyclin-dependent kinase inhibitor 1A (p21, **(F)**, and β-catenin **(G)**. Control: healthy non-CRC group (rats orally gavaged with saline); CRC groups: CR-TUM (rats injected with 1, 2-dimethylhydrazine [DMH] and left untreated); CR-TUM + FU5 (DMH-induced rats treated with 5-fluorouracil, 50 mg/kg/week, intraperitoneal, for 8 weeks); CR-TUM + LIP-MLT (DMH-induced rats treated with nanoformulated liposomal melittin, 1mg/kgBW/day/ i. p, intraperitoneal, for 8 weeks); CR-TUM + NsCR (DMH-induced rats treated with nanoformulated niosomal chrysin, 50 mg/kg, orally, for 8 weeks); and CR-TUM + LIP-MLT/NsCR (DMH-induced rats treated with liposomal melittin, 1 mg/kg BW/day/ i. p, plus niosomal chrysin, 50 mg/kg, orally, for 8 weeks) as combined therapy. Values are expressed as mean ± SE (n = 3/group); italic superscript letters denote Tukey HSD groupings, and an asterisk (*) indicates a significant difference versus the untreated CR-TUM group (p < 0.05).

### 3.6 Tipping the balance toward cell death: Modulation of apoptotic and antiapoptotic gene expression by LIP-MLT/NsCR

As shown in Figure 7, apoptosis was markedly suppressed in the DMH-induced CRC group (CR-TUM), as evidenced by the downregulation of the pro-apoptotic genes Caspase-3 (1.5-fold), Caspase-8 (2.6-fold), *BAX* (2.1-fold), and p53 (3.0-fold) relative to the pronounced activation achieved after treatment, together with the concurrent upregulation of the antiapoptotic genes c-FLIP (5.9-fold), *Bcl-2* (7.0-fold), and Survivin (1.4-fold). This imbalance favors evasion of programmed cell death and supports the uncontrolled proliferation observed in the untreated tumor tissue. Treatment with LIP-MLT or NsCR monotherapy effectively reversed this pattern by restoring the intrinsic apoptotic machinery. Both monotherapies strongly upregulated *p53*, *BAX*, Caspase-3, and Caspase-8, consistent with activation of the mitochondrial (intrinsic) apoptotic pathway, while suppressing *c-FLIP*, *Bcl*-2, and Survivin. The combined LIP-MLT/NsCR regimen was the most efficient, producing the highest pro-apoptotic expression among all groups, Caspase-3 (8.4-fold), Caspase-8 (7.4-fold), *BAX* (8.9-fold), and *p53* (6.8-fold) alongside the greatest reduction in the antiapoptotic mediators *c-FLIP* (3.2-fold), *Bcl*-2 (2.4-fold), and Survivin (0.62-fold). These changes consistently exceeded those obtained with either monotherapy or 5-FU, whose pro-apoptotic induction (e.g., Caspase-3, 8.0-fold; *p53*, 6.3-fold) was accompanied by comparatively weaker suppression of the antiapoptotic genes.

**FIGURE 7:**
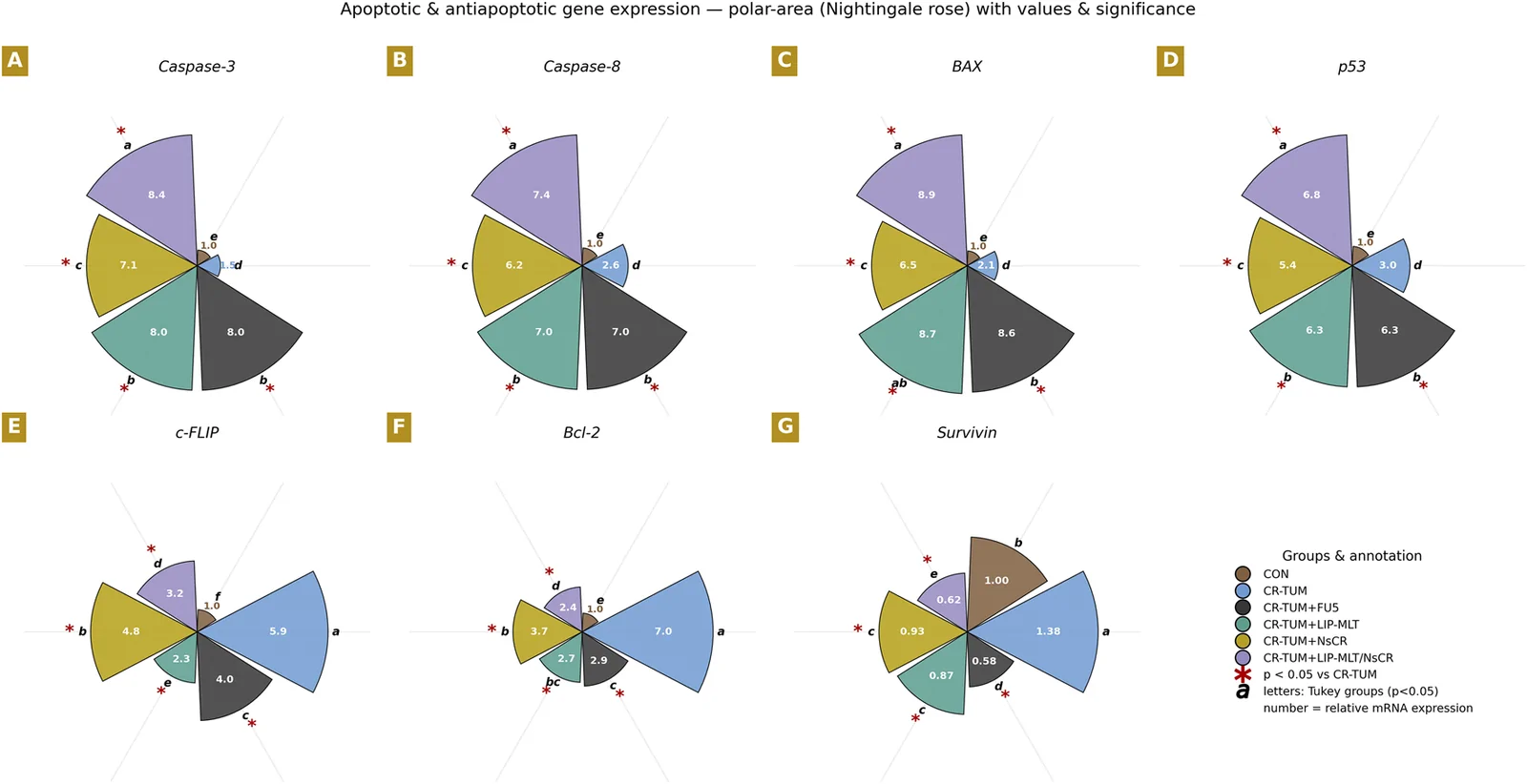
Robust modulation of apoptotic and antiapoptotic gene expression by nanoengineered liposomal melittin and niosomal chrysin (LIP-MLT/NsCR) in DMH-induced colorectal cancer, presented as polar-area (Nightingale rose) plots. In each panel, every group is drawn as a filled petal whose radius is proportional to the relative mRNA expression (value shown on the petal): caspase-3 (Caspase-3, **(A)**, caspase-8 (Caspase-8, **(B)**, BCL2-associated X protein (BAX, **(C)**, tumor protein p53 (p53, **(D)**, cellular FLICE-like inhibitory protein (c-FLIP, **(E)**, B-cell leukemia/lymphoma 2 protein (Bcl-2, **(F)**, and survivin (Survivin, **(G)**. Control: healthy non-CRC group (rats treated with saline); CRC groups: CR-TUM (rats injected with 1, 2-dimethylhydrazine [DMH] and left untreated); CR-TUM + FU5 (DMH-induced rats treated with 5-fluorouracil, 50 mg/kg/week, intraperitoneal, for 8 weeks); CR-TUM + LIP-MLT (DMH-induced rats treated with nanoformulated liposomal melittin, 1mg/kgBW/day/ i. p, for 8 weeks); CR-TUM + NsCR (DMH-induced rats treated with nanoformulated niosomal chrysin, 50 mg/kg, orally, for 8 weeks); and CR-TUM + LIP-MLT/NsCR (DMH-induced rats treated with liposomal melittin, 1 mg/kg BW/day/ i. p, intraperitoneal, plus niosomal chrysin, 50 mg/kg, orally, for 8 weeks) as combined therapy. Values are expressed as mean ± SE (n = 3/group); italic superscript letters denote Tukey HSD groupings, and an asterisk (*) indicates a significant difference versus the untreated CR-TUM group (p < 0.05).

### 3.7 Assessment of cGAS–STING innate-sensing, mTOR, and angiogenesis/metastasis gene expression in response to treatment with LIP-MLT/NsCR

The transcriptional profile of the cytosolic DNA-sensing *cGAS*–*STING* axis was markedly perturbed following CRC induction (Figures 8A–D). Relative to the control, the DMH-induced CRC group (CR-TUM) exhibited pronounced downregulation of *cGAS* (0.55-fold), *STING* (0.45-fold), and IRF3 (0.85-fold), a transcriptional pattern that is compatible with, but does not by itself demonstrate, reduced innate DNA-sensing capacity. In contrast, TBK1 mRNA was strongly upregulated in the CR-TUM group (7.0-fold vs. control); because TBK1 activity depends on phosphorylation rather than transcript abundance, the functional significance of this increase cannot be inferred from mRNA data alone. Treatment with LIP-MLT or NsCR monotherapy partially reversed these alterations, restoring *cGAS*, *STING*, and *IRF3* expression toward control levels while attenuating the elevated *TBK1* transcript. The combined LIP-MLT/NsCR regimen produced the most complete transcriptional normalization of these genes, returning *cGAS* (0.90), *STING* (0.82), and *IRF3* (0.88) close to control values and lowering *TBK1* (3.0-fold) more effectively than either monotherapy or 5-FU (p < 0.05). A parallel pattern was observed for the growth- and angiogenesis-associated genes (Figures 8E–G). In the CR-TUM group, *mTOR* (12.2-fold), *MMP9* (7.4-fold), and *VEGF* (7.7-fold) were all markedly upregulated relative to control, driving uncontrolled proliferation, extracellular matrix degradation, and tumor vascularization. Elevated *VEGF* enhances neoangiogenesis and nutrient supply, increased *MMP9* promotes invasion and metastasis, and sustained *mTOR* signaling supports tumor-cell growth and survival. Both LIP-MLT and NsCR monotherapy significantly counteracted these oncogenic changes by suppressing *VEGF*, inhibiting *MMP9*, and downregulating *mTOR*. Notably, the CR-TUM + NsCR group retained comparatively higher *MMP9* (5.0-fold) and *VEGF* (4.5-fold) than the venom-containing arms, whereas the 5-FU group achieved only intermediate suppression. Across all seven genes, the combined LIP-MLT/NsCR therapy was consistently the most effective, yielding the greatest reduction in *mTOR* (3.6-fold), *MMP9* (2.3-fold), and *VEGF* (2.2-fold) among all treated groups, alongside the strongest reactivation of the *cGAS*–*STING*–*IRF3* sensing pathway.

**FIGURE 8:**
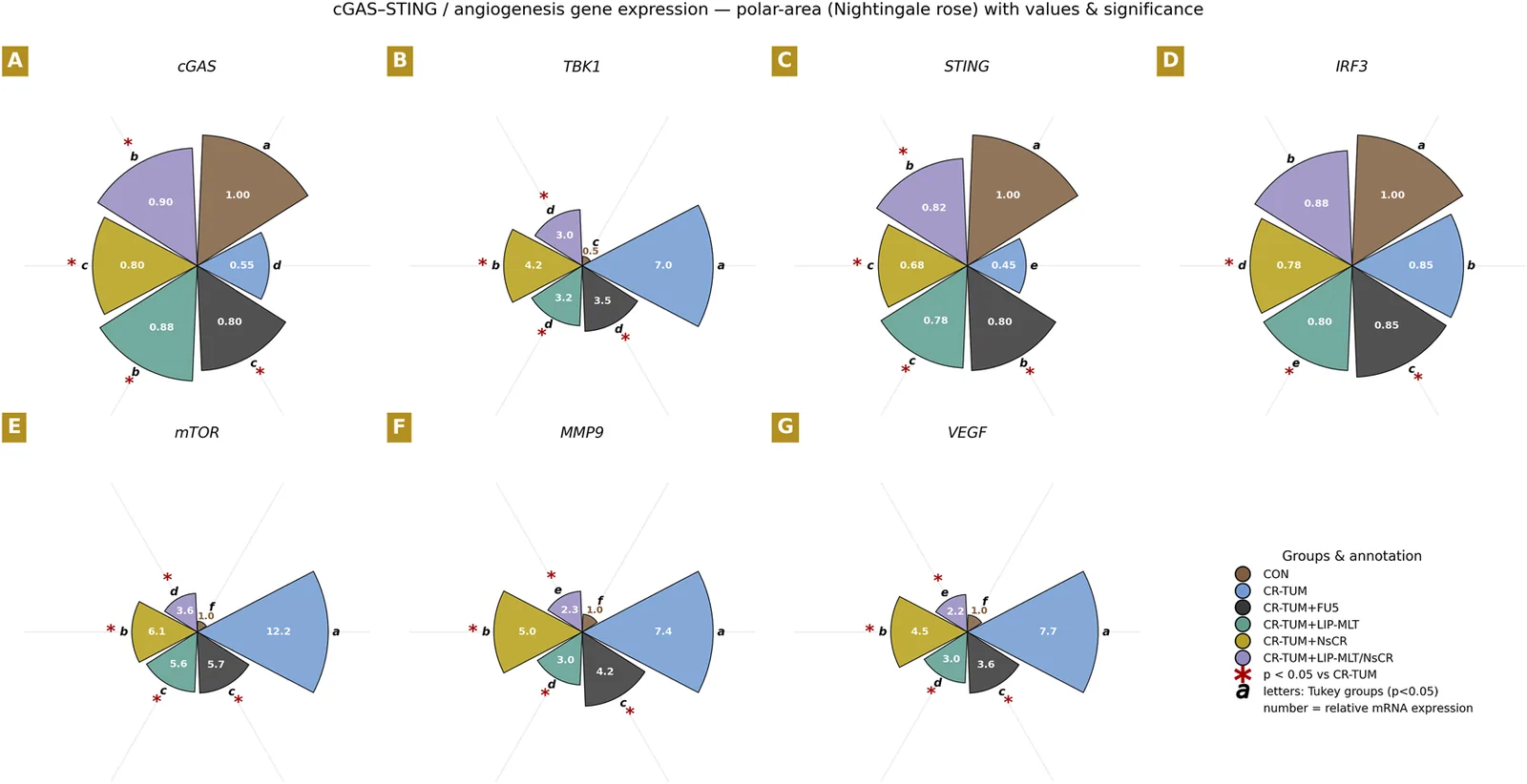
Relative mRNA expression of cGAS–STING innate-sensing genes and of growth, angiogenesis, and metastasis mediators after treatment with nanoengineered liposomal melittin and niosomal chrysin (LIP-MLT/NsCR), presented as polar-area (Nightingale rose) plots. In each panel, every experimental group is drawn as a filled petal whose radius is proportional to the relative mRNA expression (the value is printed on each petal): cyclic GMP–AMP synthase (cGAS, **(A)**, TANK-binding kinase 1 (TBK1, **(B)**, stimulator of interferon genes (STING, **(C)**, interferon regulatory factor 3 (IRF3, **(D)**, mechanistic target of rapamycin (mTOR, **(E)**, matrix metalloproteinase-9 (MMP9, **(F)**, and vascular endothelial growth factor (VEGF, **(G)**. Control: healthy non-CRC group (rats treated with saline); CRC groups: CR-TUM (rats injected with 1, 2-dimethylhydrazine [DMH] and left untreated); CR-TUM + FU5 (DMH-induced rats treated with 5-fluorouracil, 50 mg/kg/week, intraperitoneal, for 8 weeks); CR-TUM + LIP-MLT (DMH-induced rats treated with nanoformulated liposomal melittin, 1mg/kgBW/day/ i. p, intraperitoneal, for 8 weeks); CR-TUM + NsCR (DMH-induced rats treated with nanoformulated niosomal chrysin, 50 mg/kg, orally, for 8 weeks); and CR-TUM + LIP-MLT/NsCR (DMH-induced rats treated with liposomal melittin, 1 mg/kg BW/day/ i. p, plus niosomal chrysin, 50 mg/kg, orally, for 8 weeks) as combined therapy. Values are expressed as mean ± SE; italic superscript letters denote Tukey HSD groupings, and an asterisk (*) indicates a significant difference versus the untreated CR-TUM group (p < 0.05).

### 3.8 Network-referenced comparison of treatments against the untreated tumor identifies the most effective therapy

To determine which regimen most effectively reverses the malignant transcriptional program, all groups were referenced to the untreated tumor (CR-TUM) and mapped onto the gene–gene co-regulation network, in which the 31 genes segregate into a pro-tumor driver module and a tumor-suppressive/pro-apoptotic module (Figure 9). With CR-TUM set as the neutral baseline, the healthy control (CON) defined the ideal reversal pattern, characterized by strong downregulation of the entire pro-tumor module and upregulation of the suppressive module relative to the tumor. Each treatment was then evaluated by how closely its network-wide pattern reproduced this healthy-control reversal. All treatments shifted the network in the therapeutic direction, suppressing the pro-tumor driver module (red nodes) while inducing the tumor-suppressive/pro-apoptotic module (green nodes), but the magnitude and completeness of this shift differed among regimens. The effect was weakest for 5-FU, intermediate for NsCR and LIP-MLT monotherapies, and greatest for the combined LIP-MLT/NsCR therapy, which produced the most saturated node colors, the largest node sizes, and the highest proportion of genes reaching significance versus CR-TUM (p < 0.05). Quantifying this as the concordance of each group’s network-reversal profile with that of the healthy control (CON = 100%) yielded a clear ranking of therapeutic effectiveness: LIP-MLT/NsCR (64%) > LIP-MLT (58%) > NsCR (56%) > 5-FU (52%). Collectively, this tumor-referenced network analysis demonstrates that, relative to the untreated tumor, the combined LIP-MLT/NsCR regimen most completely dismantles the pro-tumor co-regulation module and restores the tumor-suppressive module toward the healthy state, providing an integrated, network-level rationale for its superior efficacy over either monotherapy or conventional 5-FU.

**FIGURE 9:**
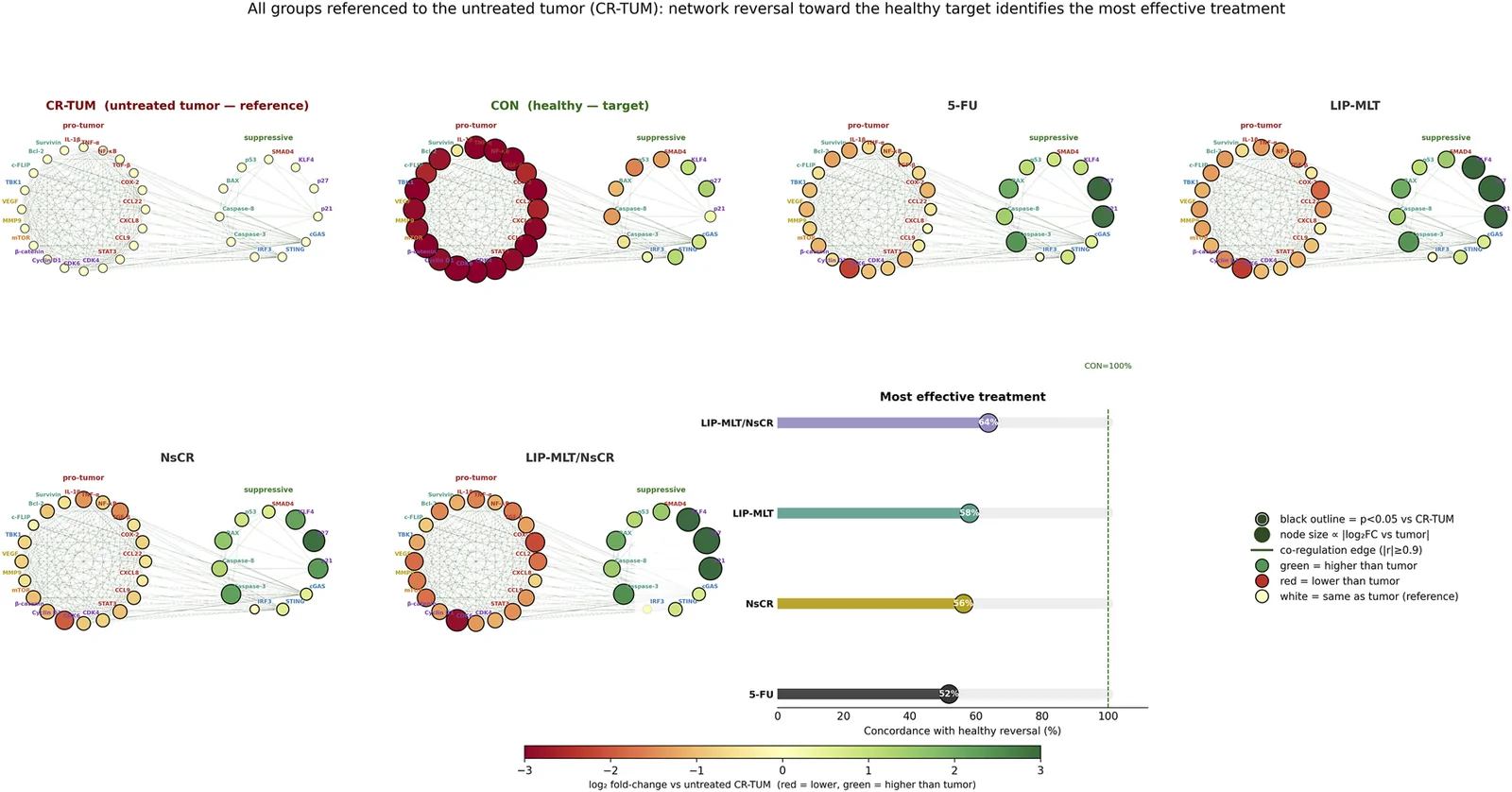
Network-referenced comparison of all experimental groups to the untreated tumor (CR-TUM) and identification of the most effective treatment. The 31 target genes spanning the six pathways (inflammatory, apoptotic/antiapoptotic, cell-cycle-arrest, cGAS–STING innate sensing, mTOR, and angiogenesis/metastasis) were organized into a gene–gene co-regulation network in which each node is a gene (label colored by pathway family) and grey edges connect strongly co-regulated gene pairs (Pearson |r| ≥ 0.9 across groups); the network resolves into two mutually anti-correlated modules, a pro-tumor driver module and a tumor-suppressive/pro-apoptotic module. In every panel, node color encodes the log_2_ fold-change of that group relative to the untreated tumor (green = higher than tumor, red = lower than tumor, white = equal to tumor) and node size is proportional to the absolute log_2_ fold-change; a black node outline denotes a significant difference from CR-TUM (p < 0.05). The untreated tumor (CR-TUM) is the neutral reference (all nodes white), and the healthy control (CON) defines the target reversal pattern. Panels: CR-TUM (reference), CON (healthy target), CR-TUM+5-FU, CR-TUM + LIP-MLT, CR-TUM + NsCR, and CR-TUM + LIP-MLT/NsCR.

### 3.9 Histopathological findings in response to treatment with LIP-MLT/NsCR

As shown in Figure 10, the healthy control group showed normal epithelial lining, mucosa, submucosa, musculosa, and serosa of the colon (Figure 10A). Meanwhile, the untreated **CR-TUM** group showed ulcerated and necrotic colitis neighboring cancerous areas, represented by hyperplastic, disorganized, and dysplastic changes in the epithelium, an increased mitotic index, and infiltration of the interstitial tissue by neoplastic cells, inflammatory cells, and hemorrhages (Figures 10B,C). Notable colitis with myositis in the musculosa and dysplasia in some colonic epithelia were observed in the **CR-TUM + FU5** group (Figures 10D,E). Additionally, the **CR-TUM + LIP-MLT** group (Figure 10F) showed maintained colon epithelium with mild exudates in the lamina propria. Remarkably, the **CR-TUM + NsCR** group demonstrated hyperplasia of the colonic glands and submucosal oedema (Figures 10G,H). Preserved colon epithelium with inflammatory cell infiltration between the glands was observed in the **CR-TUM + LIP-MLT/NsCR** group (Figures 10I,J).

**FIGURE 10:**
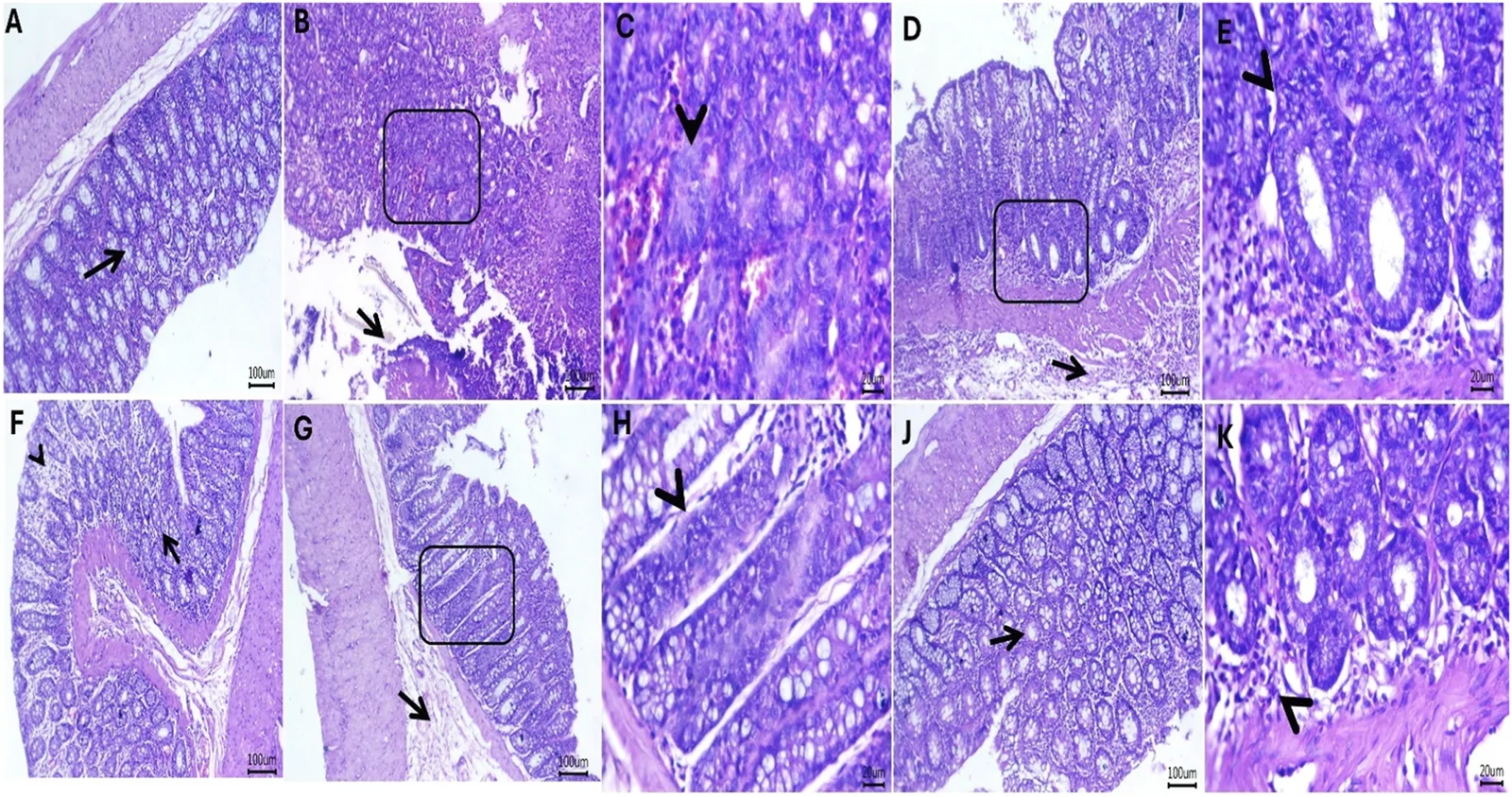
Representative photomicrographs of H&E-stained colon sections from CRC-induced groups (scale bars 100 and 20 μm). The healthy control group showed normal histological epithelial lining, mucosa, submucosa, musculosa, and serosa of the colon. **(A)** Meanwhile, the untreated CR-TUM group showed ulcerated and necrotic colitis neighbouring cancerous areas, represented by hyperplastic, disorganized, and dysplastic changes in the epithelium, an increased mitotic index, and infiltration of the interstitial tissue by neoplastic cells, inflammatory cells, and hemorrhages. **(B,C)** Notable colitis with myositis in the musculosa and dysplasia in some colonic epithelia were seen in the CR-TUM + FU5 group. **(D,E)** Additionally, the CR-TUM + LIP-MLT group **(F)** showed maintained colon epithelium with mild exudates in the lamina propria. Remarkably, the CR-TUM + NsCR group demonstrated hyperplasia of the colonic glands and submucosal oedema. **(G,H)** Preserved colon epithelium with inflammatory cell infiltration between the glands was exhibited in the CR-TUM + LIP-MLT/NsCR group. **(I,J)** Control (CON): healthy non-CRC group (rats treated with saline). CRC groups: CR-TUM (rats injected with 1,2-dimethylhydrazine [DMH] and left untreated); CR-TUM + FU5 (DMH-induced rats treated with 5-fluorouracil, 50 mg/kg/week, intraperitoneal [i.p.], for 8 weeks); CR-TUM + LIP-MLT (DMH-induced rats treated with nanoformulated liposomal melittin, 1 mg/kg BW/day, i. p., for 8 weeks); CR-TUM + NsCR (DMH-induced rats treated with nanoformulated niosomal chrysin, 50 mg/kg, orally, for 8 weeks); and CR-TUM + LIP-MLT/NsCR (DMH-induced rats treated with liposomal melittin, 1 mg/kg BW/day/i. p., plus niosomal chrysin, 50 mg/kg, orally, for 8 weeks) as combined therapy.

### 3.10 Immunohistochemical findings in response to combining treatment with LIP-MLT/NsCR

Immunohistochemical staining of VEGF protein in colon tissue revealed negative expression in the control group (Figure 11A) and overexpressing cells in the untreated **CR-TUM** group (Figure 11B). The intensity of immunoexpressed cells was less prominent in the **CR-TUM + FU5**-treated group (Figure 11C) than in the **CR-TUM + NsCR**-treated group (Figure 11D). A small number of expressing cells were noted in the group treated with **CR-TUM + LIP-MLT** (Figure 11E). No stained cells were observed in the **CR-TUM + LIP-MLT/NsCR**-treated group (Figure 11F). Moreover, immunohistochemical staining for the MMP-9 marker showed negative expression in the control group (Figure 12A), whereas strongly positive cells were observed in the untreated **CR-TUM** group (Figure 12B). Notably, cells with moderate expression were detected in the **CR-TUM + FU5**-treated group (Figure 12C). Meanwhile, few expressing cells were seen in the **CR-TUM + LIP-MLT**-treated group (Figure 12D), while only mildly expressing cells were present in the **CR-TUM + NsCR**-treated group (Figure 12E). In addition, the **CR-TUM + LIP-MLT/NsCR**-treated group demonstrated a remarkable loss of MMP-9 expression (Figure 12F).

**FIGURE 11:**
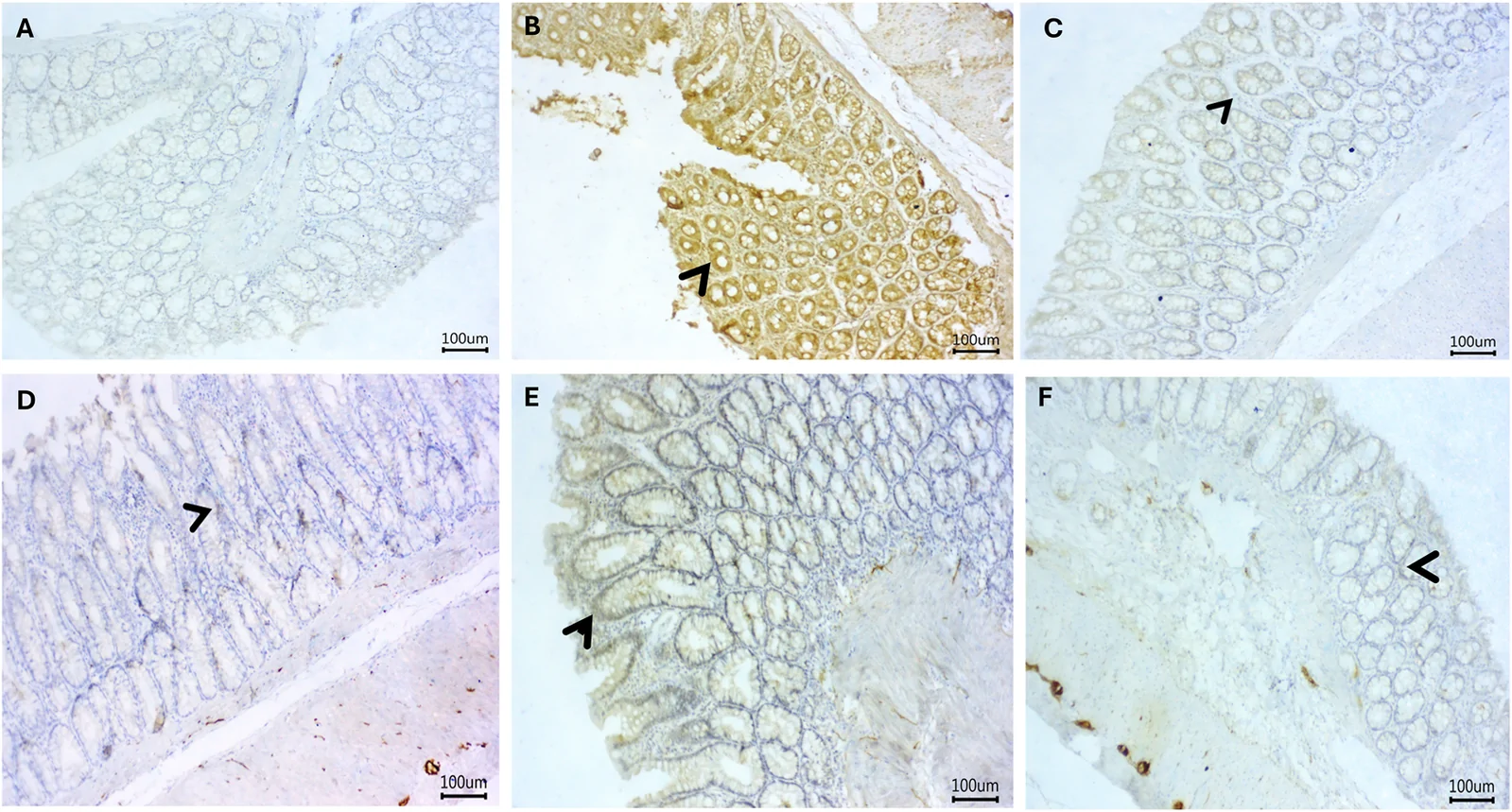
Immunohistochemical staining of VEGF protein in colon tissue revealed negative expression in the control group **(A)** and overexpressing cells in the untreated CR-TUM group. **(B)** The intensity of immunoexpressed cells was less prominent in the CR-TUM + FU5-treated group **(C)** than in the CR-TUM + NsCR-treated group. **(D)** A small number of expressing cells were noted in the group treated with CR-TUM + LIP-MLT. **(E)** No stained cells were observed in the CR-TUM + LIP-MLT/NsCR-treated group. **(F)** Control (CON): healthy non-CRC group (rats treated with saline). CRC groups: **CR-TUM** (rats injected with 1,2-dimethylhydrazine [DMH] and left untreated); **CR-TUM + FU5** (DMH-induced rats treated with 5-fluorouracil, 50 mg/kg/week, intraperitoneal [i.p.], for 8 weeks); **CR-TUM + LIP-MLT** (DMH-induced rats treated with nanoformulated liposomal melittin, 1mg/kgBW/day/i. p., for 8 weeks); CR-TUM + NsCR (DMH-induced rats treated with nanoformulated niosomal chrysin, 50 mg/kg, orally, for 8 weeks); and CR-TUM + LIP-MLT/NsCR (DMH-induced rats treated with liposomal melittin, 1 mg/kg BW/day/i. p., plus niosomal chrysin, 50 mg/kg, orally, for 8 weeks) as combined therapy. Positively stained cells appear brown, and the arrows indicate positive cells.

**FIGURE 12:**
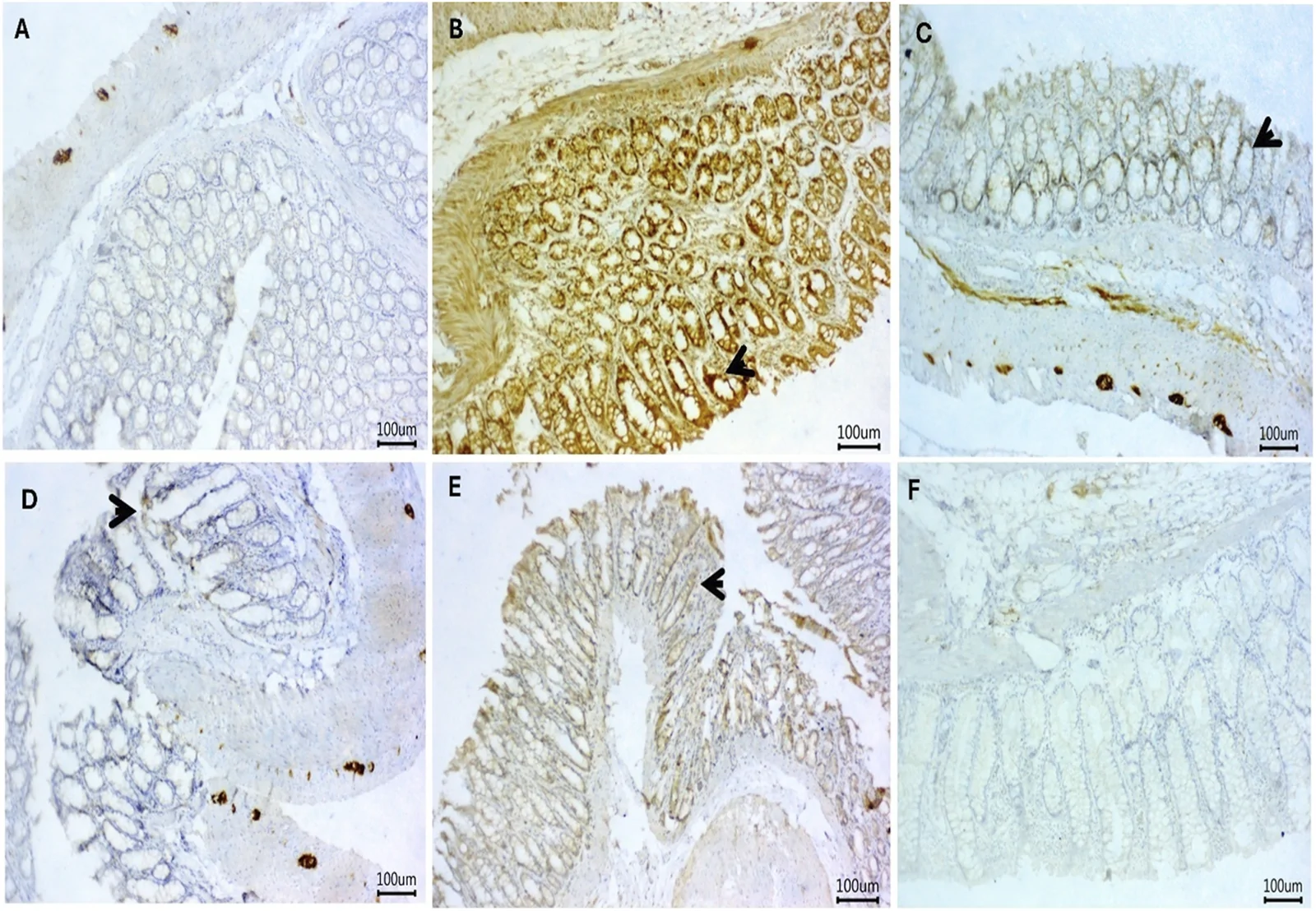
Immunohistochemical staining for the MMP-9 marker showed negative expression in the control group **(A)** whereas strongly positive cells were observed in the untreated **CR-TUM** group. **(B)** Notably, moderately expressing cells were detected in the **CR-TUM + FU5**-treated group. **(C)** Meanwhile, few expressing cells were displayed in the **CR-TUM + LIP-MLT**-treated group **(D)** while only mildly expressing cells were seen in the **CR-TUM + NsCR**-treated group. **(E)** In addition, the **CR-TUM + LIP-MLT/NsCR**-treated group demonstrated a remarkable loss of MMP-9 expression. **(F)** Control (CON): healthy non-CRC group (rats treated with saline). CRC groups: **CR-TUM** (rats injected with 1,2-dimethylhydrazine [DMH] and left untreated); **CR-TUM + FU5** (DMH-induced rats treated with 5-fluorouracil, 50 mg/kg/week, intraperitoneal [i.p.], for 8 weeks); **CR-TUM + LIP-MLT** (DMH-induced rats treated with nanoformulated liposomal melittin, 1 mg/kg BW/day/i. p., for 8 weeks); **CR-TUM + NsCR** (DMH-induced rats treated with nanoformulated niosomal chrysin, 50 mg/kg, orally, for 8 weeks); and **CR-TUM + LIP-MLT/NsCR** (DMH-induced rats treated with liposomal melittin, 1 mg/kg BW/day/i.p., plus niosomal chrysin, 50 mg/kg, orally, for 8 weeks) as combined therapy. Positively stained cells appear brown, and the arrows indicate positive cells.

## 4 Discussion

Colorectal cancer (CRC) is the second leading cause of cancer-related death worldwide. The main conventional treatment for resectable CRC is Surgical expurgation, whereas chemotherapy, immunotherapy, and radiation therapy are common treatments for unresectable CRC. The limitations of conventional therapies include severe side effects due to cytotoxicity to healthy cells and the emergence of drug resistance, in addition to lack of specificity, which might result in secondary complications (Kumar et al., 2023) that draw attention to the urgent need for novel and more effective treatment. Emerging therapeutic approaches involving natural products (animal sources), including the membrane-active peptide melittin, the principal component of honeybee (*A. mellifera*) venom, have demonstrated promising anticancer properties in various studies (Gajski, 2024; Liu et al., 2016). Moreover, the use of plant-derived active components with multi-mechanistic actions, such as chrysin, not only arrests the progression of cancer cells but also exerts a positive impact on healthy cells (Sood et al., 2024). Furthermore, incorporation of these anticancer agents into a nano-delivery system enhanced their anticancer therapeutic potential by improving controlled release, stability, and cellular uptake while minimizing toxicity (Rizkallah et al., 2025). Hence, the findings of this study highlight the significant anticancer curative role of these nano formulations, especially when employed as a combined regimen.

Rectal bleeding and weight loss are alarming features of colorectal cancer that may be accompanied by iron deficiency anemia (Olde Bekkink et al., 2010; Kuo et al., 2018). Herein, the CRC-induced group that received LIP-MLT in combination with NsCR showed remarkable restoration compared to the other treatment groups. Additionally, treatment schedules for colorectal cancer are often complicated by chemotherapy-induced diarrhea (Arbuckle et al., 2000; Kordes and Gerling, 2019). Moreover, significant levels of diarrhea regimens that contain 5-fluorouracil (5-FU) can induce diarrhea (Köhne et al., 1998), and concurrently post CRC induction, the group that received 5-FU as treatment developed a significant level of diarrhea. Notably, the greater incidence of diarrheal complications was diminished in the LIP-MLT/NsCR group, which established its efficacy in subsiding the diarheal-induced side effects of 5-FU. Subsiding of clinical signs after treatment with LIP-MLT/NsCR could be attributed to the complementary effects of antioxidant agents, combined with the membrane-active peptide melittin’s multiple antitumor functions against cancer progression. Nano-carriers protect labile melittin peptide from enzymatic degradation, improve pharmacokinetics, and enhance tumor uptake, thereby increasing antitumor efficacy (Rizkallah et al., 2025). In addition, chrysin-loaded niosomes enhance anticancer activity by boosting solubility, stability, cellular uptake, and controlled release, leading to stronger apoptosis and anti-migration effects than free chrysin in tumor cell models (Tarahomi et al., 2023).

The administration of LIP-MLT/NsCR positively impacted the overall survival rate of rats with CRC more than using each of them as single therapy or even 5-FU treatment. A greater therapeutic benefit may be attributable to the antitumor activity of LIP-MLT/NsCR, which appears to delay tumor grade escalation and disease progression, thereby substantially improving the quality of life (Luo et al., 2023). The findings reported herein on liver and renal functions, Hb concentrations, and RBC count support the inference that LIP-MLT/NsCR combinations could restore the general health condition of CRC-induced rats compared to 5-FU side effects on these monitors during cancer progression. Cancer development and response to therapy are regulated by inflammation, which either promotes or suppresses tumor progression, consistently, melittin inhibited tumor growth in CRC-bearing mice without altering biochemical or hematological parameters (Luo et al., 2023). Cancer cells express highly variable amounts of different cytokines, chemokines, and growth factors *in vitro* (Xiao et al., 2015). Inflammation accelerates colon cancer progression by sustaining DNA damage, fueling tumor cell proliferation and survival, suppressing antitumor immunity, promoting angiogenesis, and preparing pre-metastatic niches that enable spread (Tuomisto et al., 2019). Deviating from physiological microenvironmental conditions, the tumor microenvironment is dominated by several immunosuppressive cytokines (Bhat et al., 2022). The colorectal cancer (CRC) microenvironment is regulated by a complex and mutual interplay between proinflammatory and anti-inflammatory cytokine networks, the balance of which largely determines the tumor’s fate: either immune-mediated control or tumor promotion and immune evasion (Bhat et al., 2022).

In the current study, the expression of pro-inflammatory cytokines, *TNF-*α, IL-1β, iNOS, NF-κB, TGF-β1, and COX2 chemokines (CCL22, CCL9, CXC3L) was significantly upregulated in the CRC group, concurrent with increased serum levels of IL-6, IL-18, IL-17, TNF-α, IL-1β, and NF-κB. Pro-inflammatory cytokines and growth factors, including TGF-β1 and VEGF, comprise a range of intracellular signaling routes, with the STAT3 and NF-κB pathways being the most prominently engaged (Dmitrieva et al., 2016). The bidirectional relationship between inflammation and NF-κB creates a self-reinforcing cycle that promotes tumor development and progression. Inflammatory stimuli activate NF-κB, which subsequently boosts the transcription of pro-inflammatory cytokine genes, generating a self-perpetuating inflammatory cycle that promotes conditions favorable for tumorigenesis (Sadati et al., 2025) and in turn, tumor-derived cytokines reprogram a paracrine signaling network inside the microenvironment that stimulates both autonomous growth and metastatic dissemination. NF-κB is crucial for controlling inflammation and cancer, and its overactivation is linked to resistance to chemotherapy (Zhang and Yu, 2013).

Moreover, STAT3 is a master negative regulator of antitumor immunity, and persistently high expression of in CRC is associated with excessive progression and promotes cancer stemness, invasion, and angiogenesis metabolic rewiring (Rébé and Ghiringhelli, 2019) in the CRC-induced group. Accordingly, IL-6/STAT3 signaling shapes an immunosuppressive tumor microenvironment, linking chronic inflammation to immune evasion and supporting the cancer stem (Lin et al., 2020). In addition, IL-17 is widely known to be a promoter of colon cancer initiation and progression-like cells maintained by stromal IL-6, which correlates with poor outcomes (Wu et al., 2013) thus, its higher serum level in CRC supports cancer initiation.

The cytokines TNF-α and IL-6 are major mediators of CRC pathogenesis, acting through their activation of the potent oncogenic transcription factors NF-κB and STAT3, respectively, (West et al., 2015). Moreover, in the CRC-induced group, TME-mediated immunosuppressive cytokines, such as TGF-*β*, VEGF, and IL-6, play a key role in immune evasion mechanisms (Zhang et al., 2020). VEGF is a powerful angiogenic molecule that contributes significantly to cancer progression and metastasis (Ramjiawan et al., 2017). Inflammation and angiogenesis are interlinked processes, with inflammatory signals being capable of driving new blood vessel formation (Hamsa and Kuttan, 2012). Natural agents can regulate tumor angiogenesis by triggering endothelial cell death and suppressing pro-angiogenic cytokines (Kim et al., 2013; Wang et al., 2020). The mTOR pathway is a key regulatory network that controls cell growth, metabolic activity, angiogenic responses, and the maintenance of cancer cell viability (Wong, 2013). An important hallmark of advanced tumors is the upregulation of VEGF (Poon et al., 2001).

Furthermore, IL-6, a versatile immunosuppressive cytokine produced by dendritic cells, promotes immune evasion in the colorectal cancer tumor microenvironment through elaborate signaling mechanisms (Johnson et al., 2018). Furthermore, IL-6 and TNF-α synergistically stimulate STAT3 and NF-*κ*B to boost CRC cell growth [35]. TGF-*β* exhibits a dual functional role that mostly acts as a potential tumor suppressor in healthy colonic cells, and the dysregulated TGF-*β* pathway in cancer cells promotes the survival, invasion, and metastasis of CRC cells, conferring oncogenic action (Bellam and Pasche, 2010). COX-2–driven prostaglandin E2 integrates with these pathways, augmenting inflammation, immune tolerance, and epigenetic remodeling, which supports progression (Multhoff et al., 2012). CCL22 is an immunomodulatory chemokine that contributes to an immunosuppressive therapy-resistant tumor microenvironment in colorectal cancer, and its expression can be induced by microbial and macrophage cues in the colon tumor niche (Wang H et al., 2022). Additionally, knocking down of smad4 in CRC promotes colon cancer progression by upregulating the chemokine CXCL8, which recruits neutrophils to the tumor microenvironment. Consequently, SMAD4 deficiency in colorectal cancer (CRC) is linked to poorer survival outcomes, suggesting targeting the CXCL8 axis as a potential therapeutic strategy (Ogawa et al., 2019).

However, LIP-MLT, NsCR and LIP-MLT/NsCR treatments showed remarkable suppressive effects on these inflammatory biomarkers by blocking their activation at the molecular level, supporting their role in treating CRC through inhibition of pathways that attenuate inflammatory cytokine release.

Accordingly, melittin inhibits NF-κB activation, a major transcription factor involved in cellular inflammation and tumor development. The inhibition of NF-κB predicts anticarcinogenic properties and represents a key mechanism for reducing inflammation-driven cancer progression (Obeidat, 2023; Rizkallah et al., 2025). Similarly, melittin reactivates tumor-suppressive signaling and restores SMAD4 expression in colon cancer cells. This is significant because SMAD4 plays a crucial role in the TGFβ signaling pathway, which regulates proliferation and metastasis in colon cancer cells. The ability of melittin to upregulate tumor-suppressor signaling suggests that it can restore tumor suppressor function in cells where this pathway may be compromised (Nikodijević et al., 2019; Wang, 2024). By restoring SMAD4-associated signaling, melittin may restore critical tumor suppressor functions in colon cancer cells (Xu and Pasche, 2007).

Consistent with (Obeidat, 2023), melittin affects NF-κB signaling through direct inhibition of the NF-κB activation pathway and downstream suppression of pro-inflammatory gene expression, resulting in potent anti-inflammatory and anticancer effects (Rizkallah et al., 2025).

By blocking NF-κB activation, melittin prevents the transcription of genes involved in proliferation, survival, and inflammation, whereas COX-2 suppression reduces prostaglandin synthesis and inflammatory responses. These effects extend to the inhibition of VEGF and MMP expression and angiogenesis-related signaling molecules, further supporting the anticancer potential of melittin through the modulation of these interconnected pathways (El Bakary, 2020; Wang, 2024).

In agreement with our findings, chrysin reduces NF-κB activation and nuclear translocation, lowering the expression of TNF-α, IL-6, and IL-1β across cancer models, thereby dampening pro-inflammatory transcriptional programs that support tumor growth (Kurkiewicz et al., 2025). Chrysin inhibits COX-2 gene expression and prostaglandin E2 synthesis, curbing inflammation-linked proliferation, invasion, and immune suppression in tumors (Lee et al., 2024). It has been reported that triterpenoid antioxidants and other natural products can regulate apoptosis via NF-κB modulation, effectively hindering colorectal cancer development, with additional anti-inflammatory, anticancer, and anti-angiogenic activities (Naz et al., 2020). Chrysin downregulates IL-6/STAT3 signaling, a key driver of tumor-promoting inflammation and immune escape; this intersects with NF-κB to cooperatively control COX-2 and other inflammatory genes and reduced angiogenesis (Sood et al., 2024). Broader reviews summarize consistent downregulation of NF-κB, COX-2, TNF-α, IL-6, and PGE2 by chrysin and its derivatives, supporting its role as an anti-inflammatory adjunct in oncologic settings (Salari et al., 2022).

These beneficial effects on the suppression of inflammatory cascades during the progression of CRC were supported by the protection of melittin through liposomal encapsulation, reducing off-target toxicity, allowing surface functionalization to enhance anticancer delivery, improving stability loading conditions, and driving encapsulation efficiency (Rizkallah et al., 2025). Additionally, liposomal/nanoparticle melittin provoked better efficacy against cancer than its free counterpart owing to superior stability and encapsulation efficiency (Liu et al., 2016; Rizkallah et al., 2025). Moreover, Nanotherapeutic approaches enhance melittin effectiveness as anticancer agents and use lipid-coated polymeric nanoparticles as liposomes to support the functionality of melittin by acquiring high stability and biocompatibility, a long half-life *in vivo*, and a high drug-loading capacity (Rady et al., 2017). Notably, Chrysin niosomes are nonionic surfactant-based nanocarriers designed to overcome the poor solubility and bioavailability of chrysin, thereby improving tumor delivery, anti-inflammatory signaling control, and anticancer efficacy in colorectal and other cancers. (Moammeri et al., 2023). In colorectal cancer contexts, niosomal systems are highlighted as promising carriers for chemopreventive and cytotoxic agents, improving pharmacokinetics and tumor accumulation compared with free drugs (Mollazadeh et al., 2024). Also, the multiple functions of chrysin as an anticancer agent were augmented via its incorporation into nano carrier and proved to achieve good therapeutic efficacy against cancer cells (Jangid et al., 2022).

Apoptosis, which is a crucial process in maintaining cellular homeostasis, plays a paradoxical role in CRC progression. While it helps eliminate damaged cells, evasion of apoptosis allows cancer cells to thrive, gain nutrients, and avoid metabolic waste accumulation, thereby facilitating tumor growth (Sadati, Khalaji, Bonyad, Khoshdooz, Hosseini Kolbadi, Bahrami, Moeinfar, Morshedi, Ghamsaraian and Eterafi, 2025). Extensive evidence indicates that modulating apoptosis can suppress the viability and proliferation of CRC cells, hinder tumor development, and increase chemotherapy sensitivity, offering potential therapeutic benefits for colorectal cancer (Wang, 2020). In CRC, the molecular foundation of apoptosis includes complex interactions between genes and signaling pathways (Koveitypour et al., 2019). Dysfunctional apoptosis leads to prolonged survival of malignant cells. The essential genes involved in apoptosis include the tumor suppressor gene p53 and BCL-2 family, (Marei et al., 2021). In colon carcinomas, mutations in Caspase-8 can induce cell death and promote the growth of CRC, particularly in the advanced stages (Kim et al., 2003). In alignment with (Wong, 2011), following CRC induction, cancer cells can evade apoptosis through multiple mechanisms, including the downregulation of Bax and caspases-3 and -8, a phenomenon that was particularly pronounced in the untreated group in this study. As a proapoptotic Bcl-2 family member, Bax compromise mitochondrial external membrane strength via permeabilization (Er et al., 2006) and triggers cytochrome c from the mitochondria to trigger the caspase cascade (Kroemer and Reed, 2000). In cancer, proapoptotic factors such as *Bax* are suppressed, and antiapoptotic proteins such as *Bcl-2* are upregulated, promoting uncontrolled cell division (Ghobrial et al., 2005). Accordingly, apoptosis initiation in cancer cells by modifying proapoptotic proteins implicated in apoptotic pathways is an applicable approach for the treatment of cancer. Targeting the Bax/Bcl-2 balance could be beneficial for colorectal cancer interventions. Research indicates that natural compounds of this type can decrease CRC cell survival and proliferation by suppression Bcl-2 (Zheng et al., 2025). Functioning as an essential tumor suppressor, p53 orchestrates a wide range of cellular responses such as DNA repair, cell cycle control, senescence, apoptosis, differentiation, and metabolic regulation to prevent cancer formation. Colorectal carcinomas frequently exhibit loss of p53 function resulting from mutations in the gene or modifications of its regulatory mechanisms. Approximately 43% of tumors harbor P53 mutations, which in CRC are typically missense alterations that impair p53 activity, and can also specify gain-of-function properties that promote cancer cell stemness, proliferation, invasion, and metastasis. (Liebl and Hofmann, 2021). The activation of caspase-3 and subsequent degradation events probably trigger apoptosis (Khoo et al., 2010). In the CRC-induced group, cells switched to non-apoptotic cell death induced by downregulation of apoptotic genes and upregulation of antiapoptotic genes. Herein, the expression of antiapoptotic BCL-2surviving and FLIP was upregulated in the induced CRC group, indicating their important role in the chemoresistance of CRC cells.

However, treatment of rats with LIP-MLT/NsCR enhanced the expression of a death proapoptotic related genes, including Bax, caspase-3, and caspase-8 p53. However, in antiapoptotic BCL-2, FLIP and survivin activities were significantly decreased compared to the use of LIP-MLT alone or even in conventional treatment with 5-FU.

These outcomes indicated their pronounced role in the initiation of apoptosis through up-streaming and proapoptotic and conversely down-streaming of antiapoptotic gene expression.

Similarly, upregulation of *FLIP* has been found in various tumor types as antiptotic genes, a major antiapoptotic protein and an important cytokine and chemotherapy resistance factor that suppresses cytokines; its overexpression causes resistance to this apoptotic process and chemotherapy-induced apoptosis and permits cellular caspase-8 activity (Safa, 2013). In terms of colon cancer chemoprevention, flavonoids, such as chrysin, are novel natural products. There is considerable emphasis on identifying the selective induction of apoptosis and growth arrest in prostate cancer cells without cytotoxic effects in normal cells (Samarghandian et al., 2011). Flavones also have antitumor properties in cancer cells via apoptotic promotion, a process reconciled by numerous molecular pathways (Koosha et al., 2016; Sanaz Koosha et al., 2016). Chrysin suppresses *c-FLIP* expression and induces cell death in CRC cells (Ding et al., 2012). Among other apoptotic inhibitors, survivin, a novel antiapoptotic gene, is prominently expressed in CRC and several other malignancies (Asanuma et al., 2000). Survivin orchestrates the cell cycle division process and programmed cell death, thereby regulating cell cycle progression and mitosis via its role in the chromosomal passenger complex to regulate chromosome segregation and cytokinesis with antiapoptotic effect (Cao et al., 2023). Chrysin and luteolin synergistically enhanced cisplatin-induced apoptosis by upregulating proapoptotic proteins (*Bax*, caspase-8, and *p53*) and downregulating antiapoptotic proteins (*Bcl-2* and survivin), representing the therapeutic potential of these flavone combinations (Raina et al., 2023).

We attempted to further address the anticancer potential of chrysin by reducing the expression of the antiapoptotic gene, FLIP, one of the key antiapoptotic genes capable of blocking caspase-8 activity (Li et al., 2010). Chrysin induces apoptosis in cancer cells by activating the intrinsic pathway, which involves elevating proapoptotic proteins (Bax) and reducing antiapoptotic ones (Bcl-2), activating caspases (Caspase-9) and (Caspase-3), (Salari, Faraji, Jafarpour, Faraji, Rasoulpoor, Dokaneheifard and Mohammadi, 2022). It was recently shown that chrysin could induce apoptosis via induction of prosynaptic genes with significantly inhibited expression of antiapoptotic genes in T24 and induces apoptosis in association with activation of caspase-3 p53 (von Brandenstein et al., 2008). Additionally, melittin can impede the development of cancer cells and induce their death via mechanisms involving cell death and apoptosis (Luo et al., 2023; Nikodijević et al., 2019). Moreover, melittin causes apoptosis by increasing ROS and inducing cell cycle arrest in cancer cell lines (Wang, 2024; Tipgomut et al., 2018). Furthermore, melittin promotes apoptosis by decreasing the transcription of the antiapoptotic BCL2 gene and upregulating Bax and p53 (Tipgomut et al., 2018; Wang, 2024). Furthermore, upregulation of proapoptotic signaling (Bax, caspase-3, and p53) and downregulation of the antiapoptotic gene Bcl-2 were observed after treatment with melittin (El Bakary, 2020; Wang, 2024). A previous study reported that melittin downregulated the expression of the antiapoptotic gene (bcl-2) and upregulated the expression of apoptotic-related genes (Bax, p53, and caspase-3) (Nikodijević et al., 2019). Melittin’s amphipathic structure enables it to insert into the phospholipid bilayer of the cell membrane and form pores, and its cellular delivery is facilitated by encapsulation within liposomes (Duarte, 2022; Majchrzak, 2025). Additionally, encapsulating chrysin in niosomes increased apoptosis in multiple cancer models compared with free chrysin, due to better cellular uptake and endosomal escape (Jangid, Solanki, Patel, Medicherla, Pooja and Kulhari, 2022). Chrysin-loaded niosomes enhance apoptosis in cancer cells by increasing the intracellular delivery of chrysin, sustaining its release, and amplifying intrinsic apoptotic signaling pathways compared with free chrysin (Moammeri et al., 2023). Chrysin triggers the intrinsic pathway by elevating Bax, releasing cytochrome c, and activating caspase-9/3, along with ER-stress signaling and STAT3 inhibition, which are potentiated when chrysin is nano-delivered (Xu et al., 2018). HA-niosomal chrysin formulations showed controlled release (two-phase profile) with improved cellular uptake and greater biological effects than free chrysin in targeted cell models (Sahib et al., 2025). Cell cycle arrest plays a crucial role in colorectal cancer (CRC) by preventing uncontrolled proliferation of cancer cells and hindering cell cycle progression (Sun et al., 2022). Genes that were responsible for cell cycle proliferation in the tumor microenvironment (CDK4, CDK6, KLF4, cyclin D1, B-catenin, P27, and P21) were prominently upregulated in CRC that received no treatment. Accordingly, KLF4 reduced the proliferation of CRC cells by upregulating p21 and suppressing the expression of cyclin D1 (Ma et al., 2017). As an important cell cycle regulator, KLF4 influences both G1/S and G2/M checkpoints by promoting the transcription of cell cycle inhibitors and restricting the production of macromolecules, such as DNA and proteins (Whitney et al., 2018). Excessive KLF4 expression in colonic adenocarcinoma cells decreases cyclin D1 mRNA and protein levels by blocking its stimulator (Shie et al., 2000). In addition, it generates cell cycle arrest via KLF4-dependent initiation of p21 expression (Jie et al., 2017). The β-catenin pathway is critical for orchestrating cell proliferation, differentiation, invasion, and migration, and its contribution to cancer has been well documented. Evidence suggests a strong connection between β-catenin activity and the progression of colorectal cancer (Zheng et al., 2025). It is probable that mutations in cyclin-dependent kinases (CDKs) emerge throughout the initiation and progression of colorectal cancer, contributing to tumor development (Zhang et al., 2019). The high expression of Cyclin D1 in colon tumors indicates that dysregulated cyclin D1 levels drive cell cycle regulation at the initial stages of neoplasia, thus facilitating the transition from benign adenomas to invasive carcinomas (Arber et al., 1996). Cyclin D1 is a member of the cyclin family that regulates the transition of cells from the G1 to S phase (Rubin et al., 2020). Strong cyclin-dependent kinase inhibitor p21 is one considerable monitor that hinders the G2/M phase and decreases the activity of CDK1.

However, treatment with LIP-MLT/NsCR halted tumor growth and cancer cell proliferation by modulating cell cycle checkpoint genes, compared to other single treatment and 5FU treatment. Therapeutic treatment with melittin triggered cancer cell cycle arrest in the G1 phase by inhibiting cyclin-dependent kinase 4 (CDK4) and cyclin D1 and upregulating the cell cycle inhibitors p21 and p27, which is consistent with (Obeidat, 2023; Tipgomut et al., 2018). Melittin exposure can activate p53, which transcriptionally induces p21, a CDK inhibitor that halts G1/S progression; elevated p53/p21 promotes hypophosphorylated pRb, and blocks E2F-driven S-phase entry (Lee et al., 2009). In the current study, in parallel with the apoptotic activity of LIP-MLT/NsCR, Induction of p53 can upregulates P21 or cyclin-dependent kinases inhibitor 1 A (CDKN1A), P21 inhibits the activity of cyclin D1, which is in agreement with (Pasz-Walczak et al., 2001), and this finally controls cell cycle regulation, cellular senescence, and stem cell aging in colon cancer cells (Jones et al., 2005). This study demonstrated that melittin downregulates cyclin D1, triggering cell-cycle checkpoint arrest and strong cell cycle inhibition in colon cancer cells (Obeidat, 2023; Wang, 2024), and thus inhibiting cancer cell development. Accordingly, chrysin can suppress carcinogen induction of the β-catenin signaling pathway (Chen et al., 2025). Similarly, triterpenoid extract treatment significantly inhibited tumor growth (p < 0.01), and reduced tumor weight and volume, which correlated with reduced nuclear localization and upregulation of β-catenin (Shuwei et al., 2024). Evidence demonstrates that chrysin suppresses melanoma cell proliferation through p21-mediated G1 phase cell cycle arrest in A375 cells (Bhadra et al., 2012) and G2 phase arrest in esophageal squamous carcinoma (Khoo et al., 2010), indicating a complementary influence of chrysin on cell arrest. Accumulating evidence has shown that chrysin inhibits cancer cell growth by inducing apoptosis and controlling cell cycle arrest, primarily in the G1 phase (Geng et al., 2022). Mechanistic investigations have indicated that flavonoids induce apoptosis in cancer cells by targeting the Wnt/β-catenin signaling pathway, downregulating the antiapoptotic protein survivin, and blocking the nuclear translocation and transcriptional activity of β-catenin (Jearawuttanakul et al., 2020). Recent studies have shown that melittin induces apoptosis, releases cytochrome c, and causes cell cycle arrest (Tipgomut et al., 2018; Wang, 2024). Interestingly, melittin was capable of arresting the cell cycle at multiple phases, including G0/G1 and G2/M (Gajski, 2024; Majchrzak, 2025). Additionally, melittin has been found to inhibit the metastasis and invasion of cancer cells and activate intracellular pathways that ultimately result in apoptosis and cell cycle arrest (Wang, 2024).

Thus, in the current study, in light of its tumor-suppressive activity, induction of KLF4 in combination therapy comprising both LIP-MLT/NsCR -treated groups represents a potential approach for CRC therapy. (Lee et al., 2021). In the present study, the combination of NsCR and LIP-MLT upregulated p21 expression. Increased p21 expression inhibits cyclin expression, with this complex controlling the G2/M phase. Hence, the results of the present study implied that the combination of NsCR and LIP-MLT induced G2/M phase arrest via the inhibition of cdc2 and cyclin B1 by p21 upregulation in AGS/FR cells. Furthermore, the augmented role of melittin was attributed to its encapsulation with the lipid bilayer from liposomes, which intensified cell cycle arrest in cancer cells, primarily enriching G0/G1 (and in some cases G2/M) by modulating key checkpoint regulators such as p21, p27, cyclins/CDKs, and pRb, with upstream influence from p53 (Majchrzak, 2025). Additionally, liposomes may acquire melittin properties such as being more resistant to pH, light, temperature, enzyme, redox, and magnetic external stimuli, together with their active site-precise functionalization, which have significantly augmented the specificity and therefore decreased the toxicity of the encapsulated compound by allowing controllable release of multi-drug encapsulation consequential in clinically favorable biodistribution profiles and lessened non-specific uptake (AlSawaftah et al., 2021). In addition, encapsulation of natural products with liposomes enhances their anticancer effectiveness via increased surface modification, which improves the penetration of the therapeutic moiety into targeted tissues (De et al., 2018). The cyclic GMP–AMP synthase–stimulator of interferon genes (cGAS–STING) axis is a central cytosolic DNA sensor that links genomic instability to innate antitumor immunity (Shen et al., 2025). In colorectal cancer (CRC), this pathway exerts context-dependent, dual functions: acute activation drives type I interferon responses and immune surveillance, whereas its dysregulation fosters an immunosuppressive, metastasis-permissive microenvironment (Li et al., 2025; Yue et al., 2025). In the present study, the untreated DMH group (CR-TUM) displayed marked downregulation of *cGAS*, *STING*, and the downstream effector *IRF3*, together with an elevated TBK1 transcript, a pattern that may reflect impaired DNA sensing during tumor progression (Shen et al., 2025), although this interpretation requires protein-level confirmation. Treatment with LIP-MLT or NsCR restored cGAS, STING, and IRF3 transcripts toward control levels and normalized *TBK1*, and the combined LIP-MLT/NsCR regimen produced the most complete normalization of these transcripts. Restoring cGAS–STING competence has been reported to enhance antitumor responses in CRC (Li et al., 20205)). In parallel, the growth-promoting kinase *mTOR* was strongly upregulated in the CR-TUM group. Aberrant PI3K/Akt/mTOR activation is among the most frequent alterations in CRC, driving proliferation, survival, and therapy resistance (Leiphrakpam, 2024). Both monotherapies suppressed *mTOR*, consistent with evidence that natural bioactive compounds inhibit CRC through this axis (Demir et al., 2024), and the combination achieved the greatest reduction. Melittin similarly downregulates PI3K/Akt and NF-κB signaling while triggering mitochondrial apoptosis (Rady et al., 2017), providing a mechanistic basis for the venom-containing arms.

Several hypothetical mechanisms, which recommended for future study, could account for the transcriptional normalization of the cGAS–STING–IRF3 genes. First, melittin disrupts cellular and mitochondrial membranes, triggers mitochondrial ROS bursts, and activates Bax-dependent mitochondrial apoptosis (Rady et al., 2017). Mitochondrial outer-membrane permeabilization and opening of the permeability-transition pore allow mitochondrial DNA (mtDNA) to escape into the cytosol, where it is sensed by cGAS and activates STING–TBK1–IRF3 signaling (Kim et al., 2023). The co-occurrence, in the combination group, of the highest BAX, Caspase-3, and p53 mRNA levels with the most complete normalization of cGAS, STING, and IRF3 transcripts is compatible with this possibility; however, cytosolic mtDNA, mitochondrial permeabilization, and cGAS–STING activation were not measured, so this link remains speculative. Melittin-based nanoparticles have also been reported to induce immunogenic tumor-cell death and the release of damage-associated molecular patterns, which can further support innate DNA sensing (Yu and Liu, 2021).; markers of immunogenic cell death (e.g., calreticulin exposure and HMGB1 or ATP release) were not assessed in the present study.

Second, loss of STING signaling in human colorectal carcinoma is frequent and is caused mainly by epigenetic suppression rather than by gene deletion or mutation (Xia et al., 2016). Chrysin acts as a plant-derived histone deacetylase inhibitor that alters chromatin and induces p21 expression (Bhadra, Ramaiah, Reddy, Anita Krishnan, Pushpavalli, Babu, Tiwari, Rao, Yadav and Utpal Bhadra, 2012). Chrysin may therefore help relieve the transcriptional repression of cGAS and STING, which agrees with the partial restoration of this axis by NsCR alone and with the parallel restoration of p21 and KLF4. Third, STING protects against colitis-associated colorectal tumorigenesis partly by limiting excessive intestinal inflammation (Zhu et al., 2014). By suppressing NF-κB/STAT3 signaling and restoring SMAD4, the combination may have re-established a tissue environment that permits normal innate sensing, consistent with the normalization of TBK1 mRNA. All of these proposed mechanisms are speculative and remain to be confirmed at the protein and functional levels.

The angiogenic and metastatic effectors *MMP9* and *VEGF* were likewise overexpressed in untreated tumors. MMP-9 proteolytically releases matrix-bound VEGF to drive CRC angiogenesis (Hawinkels et al., 2008), and elevated MMP-9 and VEGF correlate with advanced stage and poor survival (Araujo, 2014); accordingly, gelatinase inhibition is an established antimetastatic strategy in CRC (Shoari et al., 2025). In our model, LIP-MLT and NsCR each lowered *MMP9* and *VEGF*, with the combination most effective. Chrysin is a well-characterized flavonoid that suppresses proliferation, angiogenesis, and metastasis, partly by downregulating MMP and VEGF expression (Sood et al., 2024), supporting the NsCR contribution.

Collectively, combined LIP-MLT/NsCR therapy was associated with changes at complementary nodes: it normalized cGAS–STING–IRF3 transcript levels, reduced mTOR mRNA, and lowered MMP9 and VEGF at both the mRNA and the protein (immunohistochemical) levels. The consistent superiority of the combination over either monotherapy or 5-FU indicates enhanced combined efficacy, which may reflect complementary actions whereby melittin’s membrane-active, pro-apoptotic actions (Rady et al., 2017) and chrysin’s anti-angiogenic activity (Sood et al., 2024) converge to limit DMH-induced colorectal tumor progression. The incorporation of melittin in smart nano carrier as liposomes augmented its role in inhibiting specific channels highly expressed in the cancer microenvironment, which proved its antiproliferative and proapoptotic effects, while improving its delivery and reducing its off-target toxicity cells (Wang, 2024). Dietary antioxidants, which are capable of scavenging harmful oxidants and free radicals, are of great interest for preventing disease and supporting cancer treatment (Gothai et al., 2018). Chrysin exerts strong antioxidant and anticancer effects by reducing oxidative stress in the cancer microenvironment (Cho et al., 2004). Moreover, using a natural antioxidant such as chrysin together with melittin peptides in cancer therapy can support efficacy and safety by stabilizing the oxidative balance in normal tissues while enhancing multiple complementary anticancer mechanisms in tumors (Salari, Faraji, Jafarpour, Faraji, Rasoulpoor, Dokaneheifard and Mohammadi, 2022). Furthermore, solubility, accessibility, and bioavailability may limit chrysin medical use, thus improving anticancer activity of chrysin by its inclusion in nanoparticles, which augments its therapeutic approach (Jangid, Solanki, Patel, Medicherla, Pooja and Kulhari, 2022). Additionally, nanoparticle/liposomal delivery elevates ROS and apoptotic fractions in parallel with cell cycle arrest, suggesting coordinated activation of checkpoints and death pathways when melittin is nano-delivered (Rizkallah et al., 2025).

## 5 Conclusion

This study shows that the combined administration of liposomal melittin and niosomal chrysin (LIP-MLT/NsCR) markedly attenuates DMH-induced colorectal cancer in rats, outperforming either monotherapy and conventional 5-FU without its diarrhoeagenic toxicity. Relative to the untreated tumour (CR-TUM), the combination best restored body weight, colon length, hematological and hepatorenal parameters, and survival. At the molecular level, it suppressed, it suppressed NF-κB/STAT3-driven inflammation and restored SMAD4, reactivated pro-apoptotic signaling (Caspase-3/8, BAX, p53) while lowering antiapoptotic mediators (Bcl-2, c-FLIP, survivin), re-established cell-cycle control (↓CDK4/6, cyclin D1, β-catenin; ↑p21, p27, KLF4), normalized cGAS, STING, and IRF3 mRNA expression, and curbed mTOR-, MMP-9-, and VEGF-driven angiogenesis and metastasis, with a strengthened antioxidant defense. These complementary actions are consistent with a multi-target synergistic interaction, positioning the combined melittin–chrysin nanoformulation as a promising strategy for colorectal cancer that warrants further biodistribution, dose-optimization, pharmacokinetic, and clinical study.

## Data Availability

The data are not publicly available as they are part of an ongoing project. The data that supports the findings of this study are available from the corresponding author upon reasonable request.
